# miR-196a reduces mutant Huntingtin aggregates by Rad23b-mediated degradation in Huntington’s disease

**DOI:** 10.1186/s12929-026-01292-5

**Published:** 2026-09-24

**Authors:** Chih-Wei Tung, Siew Chin Chan, Yi-Ching Chen, Po-Ming Wu, Pei-Hsun Cheng, Chuan-Mu Chen, Shang-Hsun Yang

**Affiliations:** 1 Department of Animal Science and Biotechnology, College of Agriculture and Health, Tunghai University, Taichung, 407224 Taiwan; 2 Department of Physiology, College of Medicine, National Cheng Kung University, Tainan, 70101 Taiwan; 3 Institute of Basic Medical Sciences, College of Medicine, National Cheng Kung University, Tainan, 70101 Taiwan; 4 Department of Pediatrics, National Cheng Kung University Hospital, College of Medicine, National Cheng Kung University, Tainan, 70101 Taiwan; 5 Department of Life Sciences, College of Life Sciences, National Chung Hsing University, Taichung, 40227 Taiwan

**Keywords:** Huntington's disease, miR-196a, Proteomic analysis, Rad23b, Protein aggregation, Ubiquitin-proteasome system

## Abstract

**Background:**

Huntington’s disease (HD) is a neurodegenerative disorder caused by abnormal expansions of poly-glutamine repeats in the mutant Huntingtin (mHTT). The expanded proteins form pathological aggregates to disrupt neuronal functions during disease progression, suggesting the clearance of the aggregates is considered as a potential direction for development of therapy. Our previous studies have demonstrated one specific microRNA, miR-196a, downregulates mHTT aggregates and improves the pathological phenotypes in HD. However, the detailed mechanism remains unclear.

**Methods:**

Proteomic analyses and bioinformatic tools were used to identify putative miR-196a targets in HD, and the expression of one target gene, Rad23b, was manipulated in different HD models to assess its role in mHTT aggregates.

**Results:**

Here, we identify Rad23b as a key target, and miR-196a directly downregulates Rad23b to reduce mHTT aggregates and toxicity. We also show Rad23b overexpression worsens mHTT pathology, while its knockdown or knockout diminishes aggregates, primarily through the ubiquitin–proteasome system (UPS). Moreover, Rad23b interacts with mHTT aggregates via its ubiquitin-binding domains, and promotes their ubiquitination; however, Rad23b disrupts the chymotrypsin-like activity of UPS, and contributes to mHTT accumulation. In transgenic mouse models in vivo, Rad23b increases mHTT aggregates and cell death in brains, and also worsens motor dysfunction in HD transgenic mice.

**Conclusions:**

These findings demonstrate that critical role of Rad23b in miR-196a-reduced pathological aggregates, and highly suggest downregulating Rad23b or disrupting its interaction with mHTT may offer novel strategies to mitigate mHTT aggregates to delay disease progression.

**Graphical Abstract:**

**Figure:**
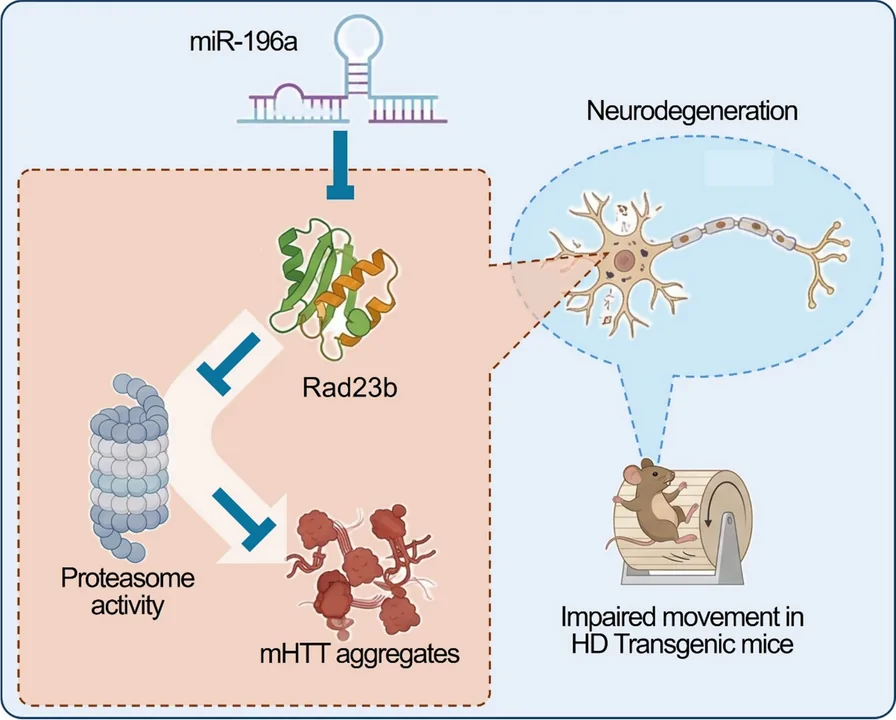

**Supplementary Information:**

The online version contains supplementary material available at https://doi.org/10.1186/s12929-026-01292-5.

## Introduction

Huntington’s disease (HD) is an inherited neurodegenerative disease with symptoms, such as dementia, involuntary movements (chorea) and behavioral changes [1]. This disease is caused by mutation of *Huntingtin* gene (*mHTT*), an abnormal increase of CAG trinucleotide repeats encoding poly-glutamine (polyQ) fragment in the first exon of *mHTT* gene, leading to the formation and misfolding of mHTT aggregates [2]. As a result, HD is also known as one of the polyQ diseases. A large body of evidence has indicated that the aggregates drive the progression of HD and disrupt various cellular functions in neurons, such as transcriptional dysfunction, transport defects, metabolic alteration, and proteasomal dysfunction [3]. These cellular dysfunctions eventually result in neuronal death, leading to pathological features, such as prominent atrophy in the striatum and cerebral cortex and associated enlargement of the lateral ventricles [4]. Therefore, elimination of mHTT is one potential therapeutical direction to alleviate HD.

microRNAs (miRNAs) are small non-coding RNA molecules that serve a critical function in regulating gene expression across various organisms, including humans. In the central nervous system, miRNAs regulate various aspects of brain functions since the genes involved in neurotransmitter receptors, transporters, and ion channels can be regulated by miRNAs [5, 6]. In addition, several miRNAs exhibit neuroprotective effects. For example, miR-27a-3p and miR-146a-5p inhibit neuronal death during intracerebral hemorrhage [7, 8], and miR-107 and miR-381 suppress neuronal damage in Alzheimer’s and Parkinson’s diseases [9, 10]. These suggest manipulating miRNA is one of directions to provide potential therapy in neuronal diseases, including HD [11]. Indeed, one miRNA, miR-196a, has been reported to play neuroprotective roles in HD. miR-196a contributes to enhance neuronal morphology through RAN binding protein 10 (RANBP10) and insulin like growth factor 2 (IGF2) pathways in different HD models [12, 13]. Furthermore, miR-196a exerts antioxidative effects via ubiquitin-specific peptidase 15 (USP15)/Nuclear factor erythroid 2 (NRF2) pathway in HD [14]. Most interestingly, miR-196a improves pathological and behavioral phenotypes in different HD models via downregulation of mHTT aggregates [15], suggesting miR-196a may regulate protein clearance pathways to degrade mHTT.

Disruption of protein clearance is known as a hallmark of HD because it leads to the accumulation of misfolded or damaged proteins in neurons [16, 17]. The ubiquitin-proteasome system (UPS) is one of the primary pathways for protein degradation in the nucleus or cytoplasm. It is responsible for the degradation of short-lived proteins, soluble misfolded or redundant proteins via 26S proteasomes [18]. The 26S proteasome is composed of two complexes, a 20S catalytic core and one or two 19S regulatory particles. The 20S core has caspase-like, trypsin-like, and chymotrypsin-like activities to cleave peptide bonds at the C-terminal of acidic, basic, and hydrophobic amino acids, respectively [19]. These catalytic activities have displayed dysfunctions in HD models. For example, significant decreases of chymotrypsin-like and caspase-like activities were also detected in most brain regions of HD patients, including striatum and cortex. These results suggest restoring or enhancing the UPS function may offer a potential target for the treatment of HD.

Rad23b, a homolog of Rad23 in yeast, was first identified as a component of nucleotide excision repair (NER) pathway, which repairs DNA damage induced by ultraviolet light and other DNA-damaging agents. Rad23 is involved in the UPS through two types of ubiquitin (Ub)-related domains. A ubiquitin-like (UbL) domain is located at the N-terminus to interact with the proteasome [20], and two ubiquitin-associated (UbA) domains play a role in recognizing ubiquitinated proteins [21, 22]. Therefore, Rad23b is considered as an Ub-binding shuttle protein and acts as a link between proteosome and ubiquitinated proteins [22, 23]. Interestingly, Rad23b accumulates in neuronal inclusions of brain tissues in HD, spinocerebellar ataxia type 3 (SCA3), SCA7, frontotemporal dementia (FTD), FTD with parkinsonism-17 (FTDP-17), amyotrophic lateral sclerosis (ALS) and Parkinson's disease patients, but the accumulation does not hamper DNA repair [24–26]. These findings implied that Rad23b may involve in UPS during the pathogenesis of different neurodegenerative diseases.

Based on our previous studies, we have shown the neuroprotective effects of miR-196a in different HD models; especially, miR-196a could significantly suppress the accumulation of mHTT aggregates. Due to that the pathological aggregates are observed in several neurodegenerative diseases and Rad23b was high throughput screened to be as a key regulator, we hypothesize that the specific target, Rad23b, of miR-196a regulates mHTT aggregate formation through the mechanisms on UPS. Our findings demonstrate that the critical role of Rad23b in miR-196a-reduced pathological aggregates, and also suggest downregulating Rad23b or disrupting its interaction with mHTT may offer novel strategies to mitigate mHTT aggregates to delay disease progression.

## Materials and methods

### Cell culture and transfection

Mouse neuroblastoma-2a cells (N2a; ATCC number: CCL-131; passage number: 182) were obtained from the Food Industry Research and Development Institute, Taiwan, and cultured in Minimum Essential Medium (Gibco, 41500-034) supplemented with 10% fetal bovine serum, 1.5 g/L sodium bicarbonate, and 1 mM sodium pyruvate. The cells were maintained in a humidified incubator at 37 °C and passaged every 2-3 days. The procedure of transfection followed the protocol provided in the manual for Lipofectamine 2000 (Invitrogen™).

### Plasmids

The constructs used in this study include GFP-84Q, mHTT-84Q, mHTT-84Q with lysine site mutations, Rad23b, HA-Rad23b, Rad23b-DsRed, shRad23b, PX459-Rad23b, pIS2-Rad23b-3’UTR, β-gal, truncated Rad23b and Ub^G76V^-GFP. The mHTT construct encodes the first exon of the human *Huntingtin* (*HTT*) gene, containing 84 CAG repeats, under the control of a human ubiquitin promoter or a cytomegalovirus (CMV) promoter. The GFP-84Q construct consists of mHTT fused to enhanced green fluorescent protein (EGFP), also driven by a human ubiquitin promoter [27]. The mHTT constructs with lysine site mutation contain single point mutations at the lysine residues located at the 6th, 9th, and 15th amino acids of mHTT, and driven by a CMV promoter. The Rad23b construct encodes the mouse *RAD23 homolog B, a nucleotide excision repair protein* (*Rad23b*) mRNA (accession number: NM_009011.4), driven by a human ubiquitin promoter. The HA-Rad23b construct includes a hemagglutinin (HA) tag fused to the 5’-end of mouse *Rad23b* mRNA. The Rad23b-DsRed construct contains a red fluorescent protein tag fused to the 3’-end of mouse Rad23b mRNA, driven by a CMV promoter. The shRad23b is a short-hairpin RNA (shRNA) targeting on *Rad23b*, and the control shRNA targets on the *luciferase* gene (shLuc), both driven by the U6 promoter. The PX459-Rad23b construct includes a single guide RNA targeting the mouse *Rad23b* gene, driven by a U6 promoter, and the coding sequence of *Streptococcus pyogenes* type II CRISPR RNA-guided endonuclease (Cas9), driven by a CMV promoter. The pIS2-Rad23b-3’UTR construct contains the 3’UTR of the *Rad23b* gene fused to a renilla luciferase reporter, driven by the SV40 early enhancer/promoter. The β-gal construct encodes the *β-galactosidase* gene under a CMV promoter. The truncated Rad23b constructs contain deletions of specific domains of Rad23b, including the ubiquitin-like domain or ubiquitin-associated domains A1, A2 or both, and are driven by a human ubiquitin promoter. The Ub^G76V^-GFP construct consists of a GFP protein fused to mutant ubiquitin, in which the glycine residue at the 76th position is substituted with valine, and is driven by a human ubiquitin promoter.

### Western blotting (WB) analysis

Cell pellets or mouse brain tissues were lysed in RIPA buffer supplemented with protease inhibitors. Protein samples mixed with 1× Laemmli sample buffer (62.5 mM Tris-Cl at pH 6.8, 2% SDS, 10% glycerol, 0.1% bromophenol blue, 300 mM beta-mercaptoethanol) were subjected for sodium dodecyl sulfate polyacrylamide gel electrophoresis (SDS-PAGE), and then transferred to an activated 0.22 μm PVDF membrane (Pall Life Sciences). The membrane was incubated with the primary antibody (mHTT, Sigma-Aldrich #MAB5374, 1:1,000; Rad23b, Proteintech #12121-1-AP, 1:1,000; Snap91, Synaptic systems #155003, 1:1,000; Larp4, Invitrogen™ #PA5-109902, 1:1,000; Rfc3, Proteiontech #11814–1-AP, 1:1,000; HA, Roche # 11867423001, 1:1,000; GAPDH, Gene Tex #GTX100118, 1:3,500; γ-tubulin, Sigma-Aldrich #T6557, 1:10,000), and subsequently incubated with an HRP-conjugated secondary antibody. Protein expression levels were detected using the T-Pro LumiLong Plus Chemiluminescent Substrate Kit (T-Pro Biotechnology, JT96-K004M) with an imaging system (Wealtec Corp.).

### Generation of Rad23b^−/−^ N2a cell line

The CRISPR-Cas9 system was employed to knock out the *Rad23b* gene in N2a cells. Simply, PX459-Rad23b plasmid was transfected into N2a cells using Lipofectamine 2000 (Invitrogen™), and single-cell colonies were isolated and expanded. Rad23b expression in different single colonies was analyzed using a Rad23b antibody via WB analysis.

### Proteomic analysis

The transfected cells were harvested in RIPA buffer supplemented with protease inhibitors, and then homogenized using an ultrasonic cell disrupter(Qsonica Q55 Sonicator). The supernatant without cell debris was mixed and washed with acetone to precipitate proteins, and the precipitated proteins were dissolved in 6 M urea (in 25 mM ammonium bicarbonate). Protein concentrations were measured using the Coomassie Bradford protein assay kit (Thermo Fisher Scientific), and 50-100 μg of protein was submitted to the NTU Consortia of Key Technologies and NTU Instrumentation Center, Taiwan, and analyzed by LC-MS/MS via Orbitrap Elite Mass Spectrometry (Thermo Scientific™). The normalized abundances from the proteomic analysis were processed using the MetaboAnalyst 5.0 platform [28]. Data scaling was performed using auto-scaling, and the volcano plot was generated from the Univariate Analysis section of the platform.

### Bioinformatic analysis

Candidates identified through proteomic analysis and selection were submitted to the Database for Annotation, Visualization and Integrated Discovery (DAVID) web tools [29]. The functional categories of these candidates were analyzed by KEGG pathway database. Bubble charts for KEGG enrichment analysis were generated using the ggplot2 package in R version 4.4.1. The putative miR-196a targets were predicted by TargetScanHuman 8.0 [30], miRDB [31], miRSystem [32], PicTar [33], and miRmap [34] websites. The expression profiling of RAD23B in HD patients and controls was conducted using data retrieved from the Gene Expression Omnibus (GEO) database (https://www.ncbi.nlm.nih.gov/geo/). The dataset utilized in this study is available under the accession number GSE194241 [35].

### Coimmunoprecipitation (Co-IP)

Cell pellets from transfected cells were fixed with 2–4% paraformaldehyde (PFA), and homogenized in RIPA buffer using an ultrasonic cell disrupter (Qsonica Q55 Sonicator). The sonicated lysates were centrifuged at 16,000 × g for 10 min at 4 °C, and protein concentration of supernatant was determined using the Coomassie Bradford protein assay kit. The proteins were mixed with equal amount of rat normal IgG, HA antibody (Roche, 11,867,423,001) or GFP antibody (GeneTex, GTX113617), and then incubated with protein G magnetic beads (GenScript, L00274) on a rotator overnight at 4 °C. The protein-antibody-coated beads were collected using a magnetic separator, boiled in 1× Laemmli sample buffer at 100 °C for 10 min, and then subjected for WB analyses.

### Immunofluorescence (IF)

The cells were fixed with 4% PFA, washed with PBS, and incubated in blocking buffer (PBS containing 5% donkey serum, 0.2% Triton X-100, 3 mM sodium azide, 0.1% saponin, and 2% BSA) for 1 h. These cells were incubated with primary antibodies, mHTT (1:50, Sigma-Aldrich, MAB5374) or Rad23b (1:200, Proteintech, 12121-1-AP), and then incubated with Alexa Fluor-conjugated secondary antibodies (Invitrogen™, Alexa Fluor 647 #A-21244, Alexa Fluor 488 #A-11034, 1:1000) and Hoechst 33342 (Invitrogen™, H1399). The fluorescent images were captured by using a DMi8 fluorescent microscope (Leica) with a Metamorph software. For the brain tissues, mice were anesthetized and perfused with cold PBS, and then brain samples were fixed in 4% PFA. Brain sections were prepared using a cryostat (CryoStar NX50 OPV) with a thickness of 20 μm, and these sections were subjected for IF following the protocol described above.

### Detection of cell death

For the cells, propidium iodide (PI) staining was used to determine cell death. Treated cells were cultured in 1 μg/mL PI and 1 μg/mL Hoechst 33342 for 30 min, and the fluorescent images were captured by using a DMi8 fluorescent microscope to evaluate percentage of cell death. For mouse brains, the dead cells were labeled using In Situ Cell Death Detection Kit TMR red (Roche). In brief, the brain sections were incubated with enzyme solution at 37 °C for 1 h after IF staining, and images were captured by using a DMi8 fluorescent microscope.

### Luciferase activity assay

N2a cells were co-transfected with miRNA, pIS2-Rad23b-3’UTR vector and β-gal constructs. After 48 h of incubation, the cells were subjected for determination of luciferase activity using the Dual-Luciferase® Reporter Assay System (Promega) and a luminometer (Berthold Detection System, Sirius). For the β-galactosidase activity, Assay Buffer (200 mM sodium phosphate, pH 7.3, 2 mM MgCl₂, 100 mM β-mercaptoethanol, and 1.33 mg/mL o-nitrophenyl-β-D-galactopyranoside) was mixed with cell samples, and the color intensity was measured at 490 nm using an ELISA reader.

### Ub^G76V^-GFP reporter assay

N2a cells were co-transfected with *Rad23b*, *mHTT* vectors and Ub^G76V^-GFP constructs (Addgene) for 48 h, and then the cell nuclei were stained using Hoechst 33342. The fluorescent images were acquired using a DMi8 fluorescent microscope, and the numbers of Ub^G76V^-GFP-postive and Hoechst-positive cells were analyzed by ImageJ software. The expression levels of Ub^G76V^-GFP were determined using a GFP antibody by WB analysis.

### Proteasome activity assay

The procedure of proteasome activity assay was adapted from a previous study [36]. The transfected cells were lysed in proteasome lysis buffer (50 mM Tris, pH 7.4, 1 mM EDTA, 2 mM ATP, and 1% Triton), and the protein concentration was determined using the Coomassie Bradford protein assay kit (Thermo Fisher Scientific). The extracted proteins were incubated with one of the fluorogenic 7-amino-4-methylcoumarin (AMC)-tagged peptide substrates, including Suc-LLVY-AMC (Enzo Life Sciences, BML-P802-0005), Boc-LRR-AMC (Enzo Life Sciences, BML-BW8515-0005) or Z-LLE-AMC (Enzo Life Sciences, BML-ZW9345-0005). The fluorogenic AMC intensity (excitation at 380 nm and emission at 460 nm) was measured using a multi-mode microplate reader (Molecular Devices, SpectraMax i3x) at 37 °C in kinetic mode, with readings taken every 5 min over a 120-min period.

### Transgenic mice

HD transgenic mice (R6/2 mice), miR-196a, and Rad23b transgenic mice were utilized in this study. HD mice express a N-terminus of the human *HTT* gene with 120-150 CAG repeats in exon 1 region [37]. miR-196a and Rad23b transgenic mice were generated by infecting FVB mouse zygotes with lentiviruses expressing miR-196a or Rad23b, respectively, via microinjection. This study was conducted in accordance with the guidelines set forth in the Guide for the Care and Use of Laboratory Animals of the National Institutes of Health. All protocols were reviewed and approved by the Institutional Animal Care and Use Committee at National Cheng Kung University, Taiwan. Mice were provided with food and water ad libitum and housed in a temperature-controlled environment (21±1 °C) with a 12-h light-dark cycle. The laboratory animal center at National Cheng Kung University has been fully accredited and certified by Association for Assessment and Accreditation of Laboratory Animal Care International (AAALAC International).

### Behavioral tests

*Hindlimb clasping test*: Mice were lifted by their tails, and the hindlimb clasping phenotypes were recorded over a 30-s observation period. The clasping score was defined according to previously established criteria [38]. Briefly, the clasping scores ranged from 0 to 3. A score of "0" indicated that the hindlimbs were splayed outward and away from the abdomen. A score of "1" was assigned when one hindlimb was retracted inward toward the abdomen for at least 50% of the observation period. A score of "2" was assigned when both hindlimb partially retracted inward toward the abdomen for at least 50% of the observation period. A score of "3" was given when both hindlimbs were completely retracted inward toward the abdomen for at least 50% of the observation period.

*Rotarod test*: Motor performance was assessed using a rotarod apparatus (Singa). Eight-week-old mice were trained for three consecutive days at a constant speed of 4 revolutions per minute (rpm) for a duration of 3 min. The mice were tested on the rotarod apparatus set to accelerate from 4 to 40 rpm over a 5-min period. A total of six trials were conducted, with a 3-min interval between each trial. The parameter of "latency to fall" was recorded for analysis.

### Imaging and analysis

IF images were acquired using a DMi8 fluorescent microscope (Leica). All subsequent analyses were performed with ImageJ software (v.1.54 g, NIH). For generating intensity plot profiles, all fluorescent images were also converted to 8-bit format. The region of interest (ROI) was defined by drawing a straight line, and this region was labeled in other images using the ROI manager. The intensity plot profiles were generated through the "Analyze > Plot Profile" function.

### Statistical analysis

Statistical analyses were performed using an unpaired Student’s t-test or one-way analysis of variance (ANOVA). The Student’s t-test was employed to compare differences between two groups, while one-way ANOVA was utilized to assess differences among three or more groups. Multiple comparisons were performed using Šídák’s multiple comparisons test. A p-value of less than 0.05 was considered statistically significant. All calculations were conducted using GraphPad Prism 6 software (GraphPad Software). All data are presented as mean ± standard deviation (SD).

## Results

### Proteomic analysis identifies putative targets of miR-196a

**Fig. 1:**
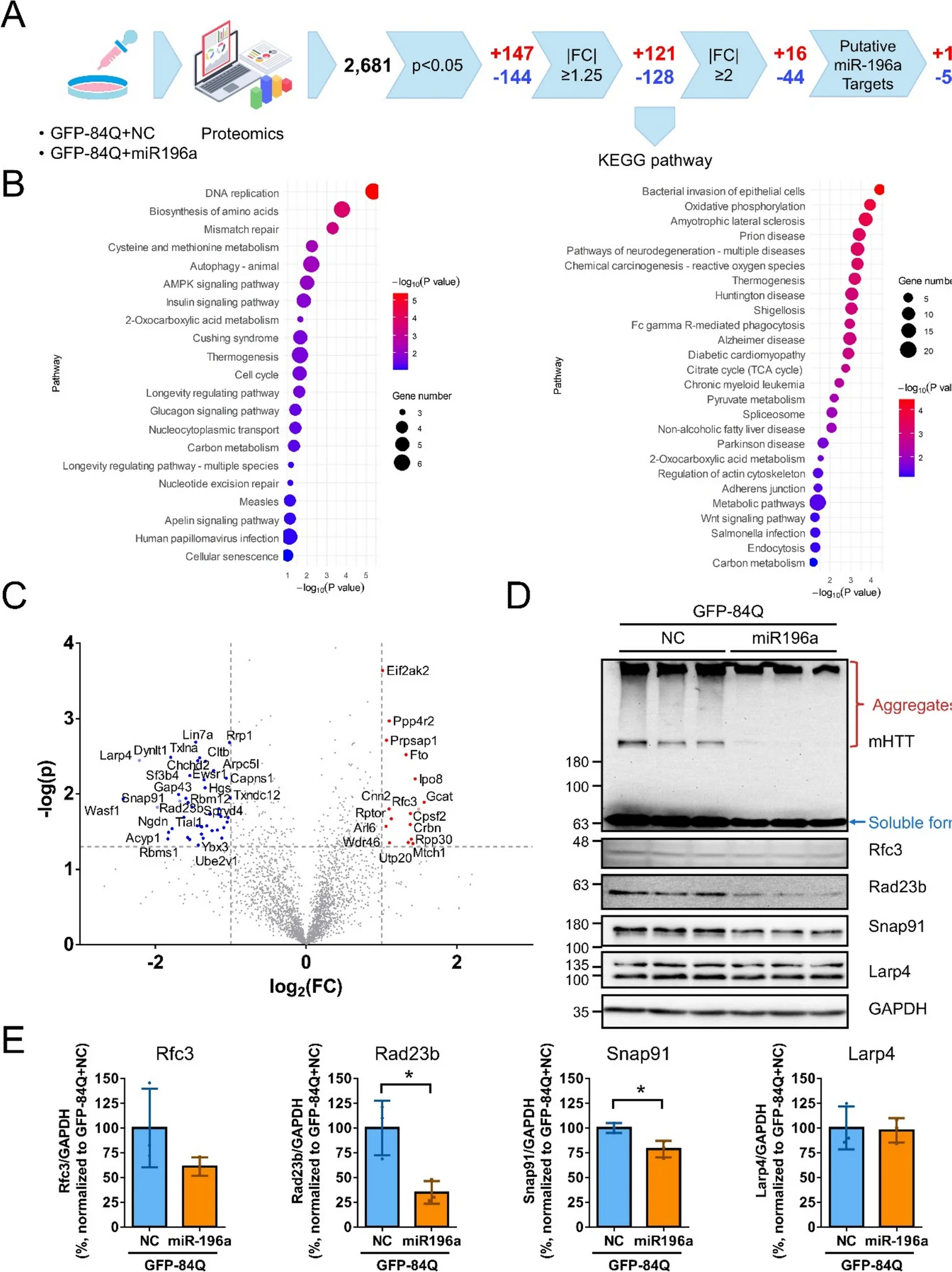
Potential targets of miR-196a are identified through proteomic analyses in HD cells. **A** The flow chart shows the screening strategies in this study. Numbers indicate the remaining candidates after screening of different parameters. Positive (red) and negative (blue) numbers represent the number of upregulated and downregulated proteins, respectively. FC, fold change. **B** KEGG enrichment analyses were performed using upregulated (left panel) and downregulated (right panel) candidates. Gene number indicates the number of differentially expressed genes enriched in each pathway. P value indicates the p-value calculated by Fisher's Exact test. **C** Volcano plot shows the expression profiling of different candidates after overexpression of miR-196a in HD cells. The threshold in the volcano plot was p<0.05 and |log_2_(fold change)|≥ 1. Red dots indicate upregulated proteins and blue dots indicate downregulated ones. **D** Representative immunoblots showed the expression of putative miR-196a targets in HD-N2a cells after transfection with a non-relevant miRNA control (NC) or miR-196a mimics for 48 h. **E** The quantitative results from (**D**) showed that Rad23b and Snap91 are downregulated by miR-196a (N=3, two-tailed Student’s t test, P for Rad23b expression=0.0194; P for Snap91 expression=0.0198). Significantly different at *p < 0.05

According to the targeting principle of miRNA, one miRNA may regulate hundreds of gene expression [39]. To efficiently identify potential molecular targets of miR-196a, N2a cells were transfected GFP-84Q with miR-196a or non-relevant miRNA control (miR-NC), and then subjected for the proteomic analysis using LC-MS/MS. As a result, 2,681 proteins were identified, and 147 proteins were significantly upregulated and 144 proteins were significantly downregulated in the GFP-84Q + miR-196a group compared to those of the GFP-84Q + miR-NC group (Fig. 1A). We further applied a fold-change cutoff, selecting proteins with expression changes greater than 1.25-fold or less than −-1.25-fold, yielding 121 upregulated proteins and 128 downregulated proteins. These altered proteins were submitted to the DAVID website for KEGG pathway enrichment analysis, which revealed significant enrichment in nucleotide-related pathways and autophagy among the upregulated proteins, while downregulated proteins were associated with neurodegenerative disease-related pathways (Fig. 1B and Supplementary Table 1 and 2). We further applied a stricter fold-change cutoff, focusing on proteins with expression changes greater than 2-fold or less than −-2-fold, and identified 60 proteins, including 16 upregulated and 44 downregulated ones (Fig. 1C). Additionally, we intersected these genes with potential targets of miR-196a using five miRNA target prediction databases (TargetScanHuman 8.0, miRDB, miRSystem, PicTar, and miRmap), and identified 6 candidates (Supplementary Table 3). Due to the availability of commercial antibodies, we validated four candidates, including Rfc3, Rad23b, Snap91 and Larp4, in N2a cells transfected GFP-84Q with miR-196a and miR-NC through WB (Fig. 1D), showing Rad23b and Snap91 were significantly downregulated by miR-196a (Fig. 1E). Given the fact that Rad23b may relate to proteasome functions which highly affect mHTT aggregates, we further focus on the role of Rad23b in miR-196a-regulatory neuroprotection in HD.

### Rad23b is directly regulated by miR-196a

**Fig. 2:**
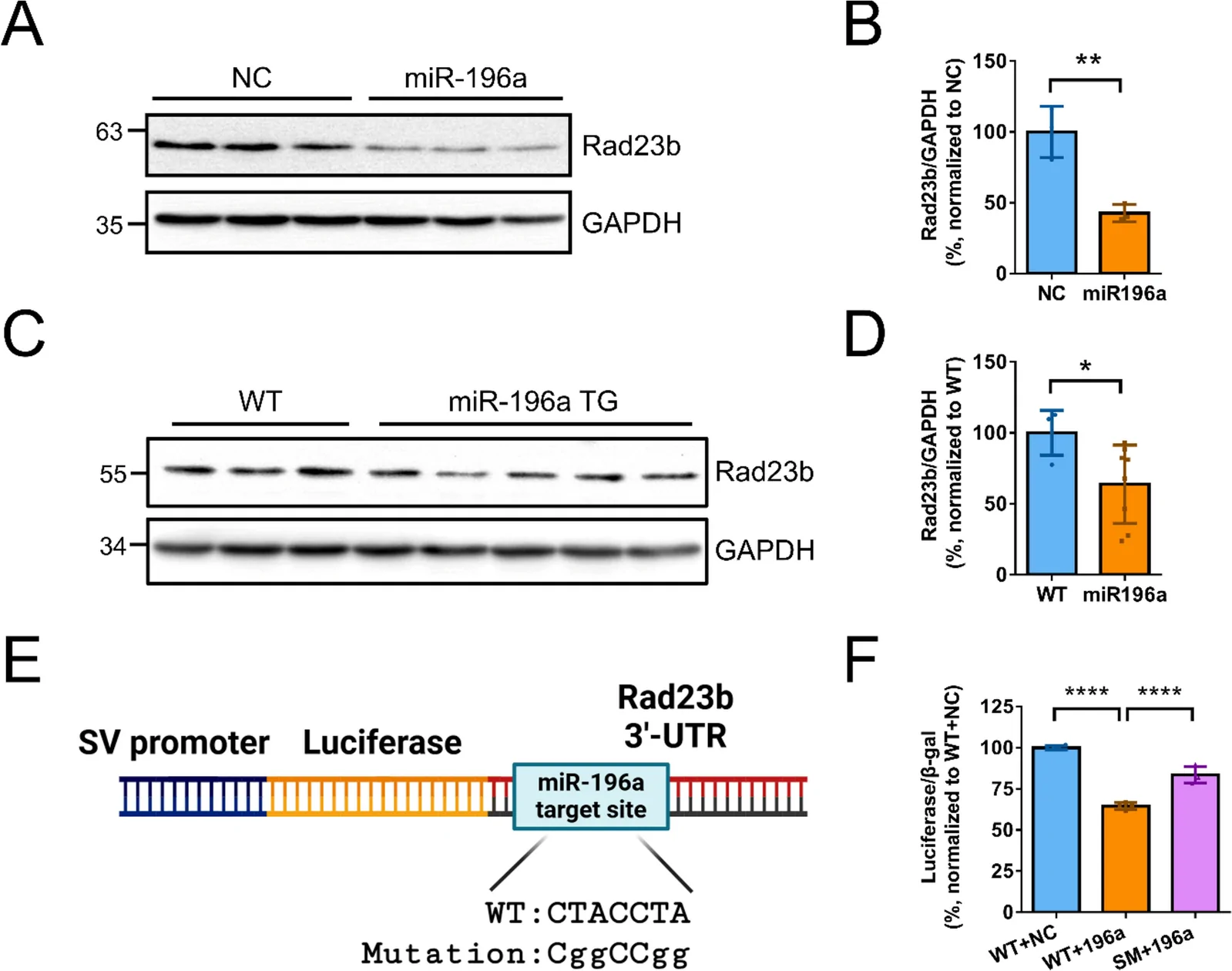
miR-196a directly downregulates Rad23b in vitro and in vivo. **A** Immunoblots show the protein level of Rad23b after transfection with miRNA NC or miR-196a. **B** The quantitative results from (**A**) indicate that miR-196a downregulates Rad23b expression (N=3, two-tailed Student’s t test, P=0.0065). Significantly different at **p < 0.01. **C** Immunoblots show the protein level of Rad23b in wild-type (WT) or miR-196a transgenic (TG) mice. **D** Quantification of Rad23b protein levels from mouse samples shows a moderate but significant decrease of Rad23b expression in cerebral cortex of miR-196a TG mice. (N for WT=4; N for miR-196a TG =8; two-tailed Student’s t test, P=0.0378). Significantly different at *p < 0.05. **E** A schematic illustrates the construction of 3’-untranslated region (UTR) of Rad23b in a reporter construct. WT, wild type; SM: site mutation. **F** Quantitative results from the luciferase assay showed that the luciferase activity is downregulated by miR-196a mimics, whereas the mutation of miR-196a target site blocks the suppression of miR-196a (N=4, one-way ANOVA). miRNA NC serves as a control treatment. GAPDH and β-galactosidase (β-gal) expression serve as internal controls. Significantly different at ****p < 0.0001

miRNA dominantly functions to suppress target genes, and the working mechanism is through directly binding to 3’UTR of target mRNA to inhibit gene translation [40]. Since we predict Rad23b is a potential target of miR-196a in Fig. 1, we first examined the expression profiling of Rad23b after miR-196a expression in vitro and in vivo. We transfected miR-196a into N2a cells, and assessed Rad23b expression via WB, showing Rad23b expression is significantly downregulated under the treatment of miR-196a (Fig. 2A, B). We also used the cerebral cortex from miR-196a transgenic mice at 3 months of age to detect Rad23b expression via WB, showing a moderate but significant reduction in Rad23b expression in the miR-196a transgenic mice (Fig. 2C, D). These results suggested that the expression levels of miR-196a and Rad23b are negatively correlated. We further examined whether miR-196a directly binds to 3’UTR of *Rad23b* to decrease Rad23b expression via the predicted binding site. We cloned the 3’UTR sequence of *Rad23b* in the downstream of a luciferase reporter, and also mutated the predicted sequence of miR-196a binding site through site-directed mutagenesis (SM; CTACCTA → CggCCgg) (Fig. 2E and Supplementary Fig. 1). As miR-196a was delivered into N2a cells with the reporter construct, the luciferase activity revealed a significant decrease in the miR-196a group compared to that in the NC group, whereas the decrease of luciferase activity was not observed in the SM reporter construct (Fig. 2F). These findings indicate that miR-196a directly regulates Rad23b expression by targeting 3’UTR of *Rad23b* mRNA.

### Rad23b elevates the mHTT aggregates

**Fig. 3:**
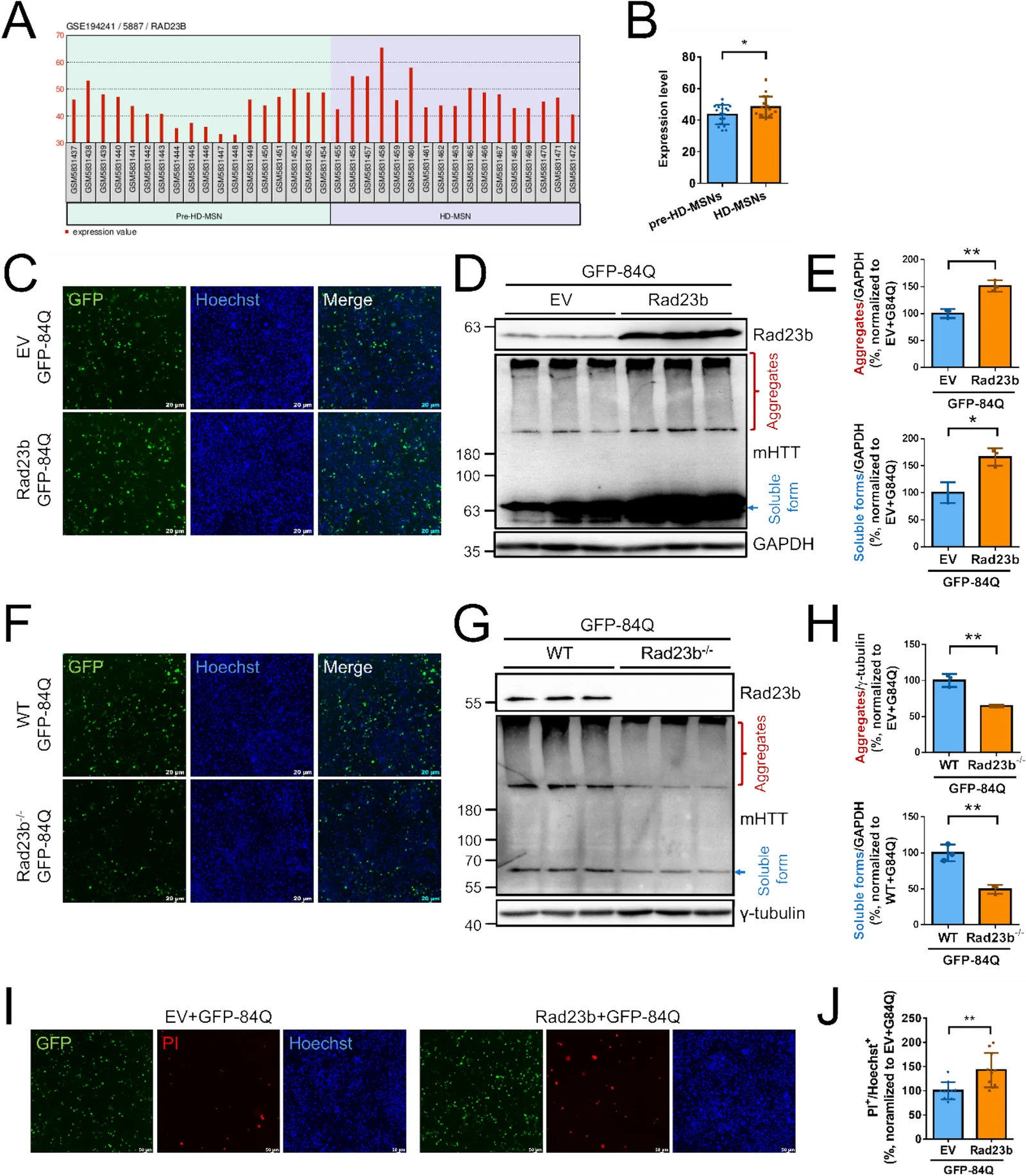
Rad23b positively corelates with the expression of mHTT aggregates. **A** The GSE194241 dataset shows the expression of Rad23b in directly reprogrammed striatal neurons derived from presymptomatic (Pre-HD-MSN, N=18) and symptomatic (HD-MSN, N=17) HD patients. **B** The quantitative results from (**A**) indicate Rad23b is upregulated in the HD-MSN group (two-tailed Student’s t test, P=0.0325). Significantly different at *p < 0.05. **C** Fluorescent images show GFP-84Q expression in N2a cells after transient transfection with Rad23b. An empty vector (EV) served as the control treatments. **D** Immunoblots show the expression of GFP-84Q after overexpression of Rad23b. **E** The quantitative results indicate that both the soluble form and aggregates of GFP-84Q are upregulated after the overexpression of Rad23b (N=3, two-tailed Student’s t test, P for aggregate expression=0.0027; P for soluble form=0.0103). Significantly different at *p < 0.05; **p < 0.01. **F** Fluorescent images show the expression of GFP-84Q in Rad23b^−/−^ N2a cells. **G** Immunoblots showed the expression of the soluble forms and aggregates of GFP-84Q in Rad23b^−/−^ N2a cells. **H** The quantitative results demonstrate that the expression of GFP-84Q is downregulated after knockout of Rad23b. (N=3, two-tailed Student’s t test, P for aggregate expression=0.0025; P for soluble form=0.0026). Significantly different at **p < 0.01. **I** Fluorescent images show the numbers of the GFP-84Q^+^ and PI^+^ cells (red). **J** The quantitative results of the PI staining assay demonstrate that cell death (PI^+^/Hoechst^+^) is elevated after the overexpression of Rad23b (N = 10, two-tailed Student’s t test, P = 0.0034). Significantly different at **p < 0.01. Nuclei were stained with Hoechst 33342 (blue)

Since Rad23b is a potential target of miR-196a, which offers neuroprotective effects in HD, we next demonstrate the effect of Rad23b in HD. We first analyzed a GEO dataset, GSE194241, which displays RNA sequencing data from directly reprogrammed striatal neurons in HD patients, and results show that a significant increase of *Rad23b* mRNA levels in HD patient-derived striatal neurons compared to those from presymptomatic HD patient-derived neurons (Fig. 3A, B), highly suggesting Rad23b may be related to HD progression. Since mHTT aggregate is one of the most important characteristics in HD [12, 14, 15], we further address the effects of Rad23b on the formation of mHTT. We co-transfected Rad23b and GFP-84Q into N2a cells, and observed severe aggregates of GFP-84Q after Rad23b overexpression (Fig. 3C). Using WB analysis, we also detected higher levels of aggregates and the soluble form of GFP-84Q after overexpression of Rad23b (Fig. 3D, E). On the contrary, we transfected GFP-84Q into Rad23b^−/−^ N2a cells, and the fluorescent aggregates were reduced in Rad23b^−/−^ cells compared to those in wild-type cells (Fig. 3F), which were supported by WB analyses in Rad23b^−/−^ cells (Fig. 3G, H) and under shRad23b treatment (Supplementary Fig.  2 A). To exclude the possibility that GFP fragments contribute to the aggregates of GFP-84Q regulated by Rad23b, we also examined the expression of mHTT-84Q, which has no *GFP* gene, after alteration of Rad23b in N2a cells, and results showed that mHTT aggregation levels were positively related to Rad23b levels (Supplementary Fig. 2B and 2 C). Given that the toxic effects of mHTT lead to neuronal death [41], we next determined cell death after Rad23b overexpression in HD cells using PI staining (Fig. 3I), showing a significant increase in the PI^+^/Hoechst^+^ ratio in the Rad23b overexpression group (Fig. 3J). These findings suggest that Rad23b exacerbates mHTT aggregates in HD cells.

### Rad23b is involved in miR-196a-suppressed mHTT aggregates

**Fig. 4:**
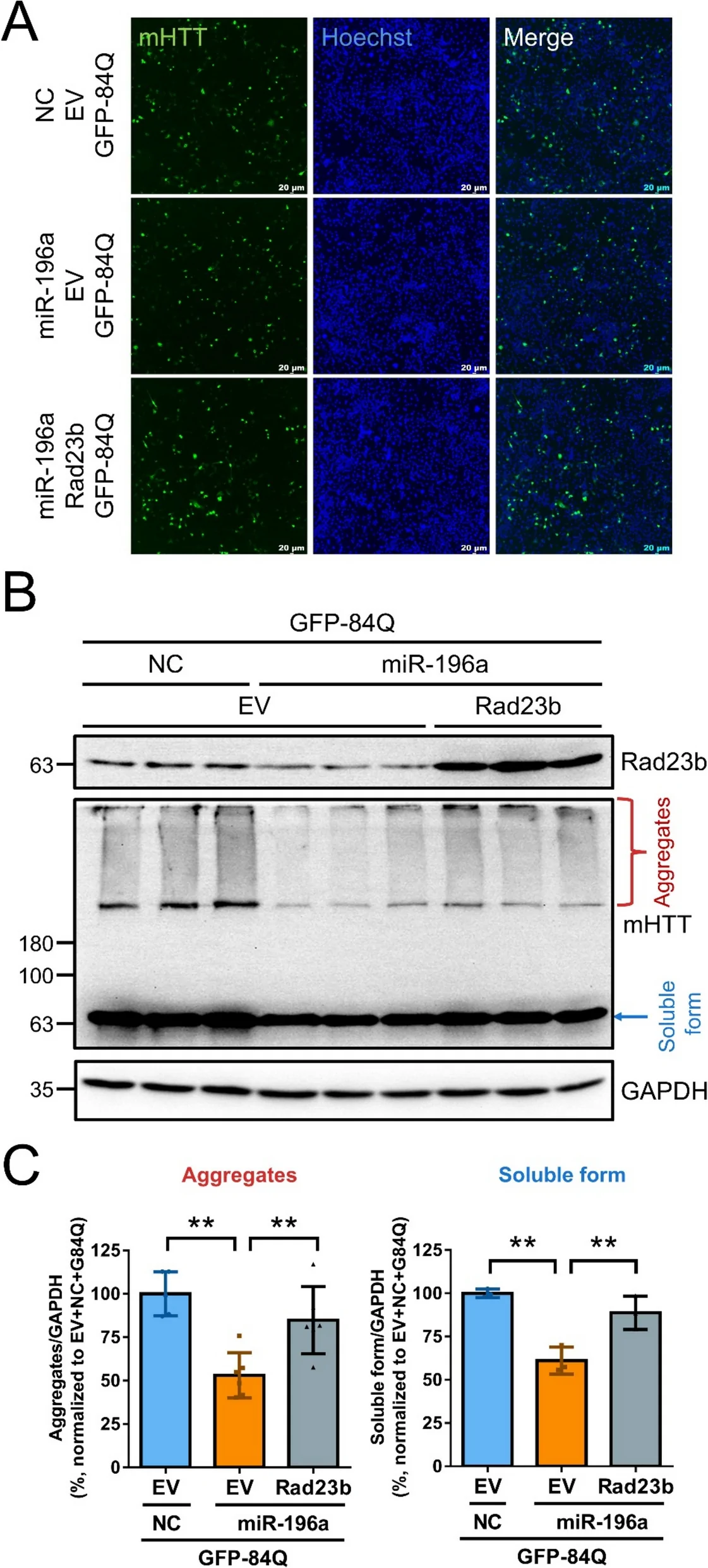
Rad23b blocks miR-196a-mediated suppression of mHTT aggregates. **A** Fluorescent images show the expression of GFP-84Q in N2a cells after transfection of different constructs as indicated. Nuclei (blue) were stained with Hoechst 33342. **B** Immunoblots show the expression of GFP-84Q aggregates after transfection of different constructs. **C** Quantitative results of GFP-84Q (G84Q) soluble forms and aggregates indicate that miR-196a-mediated suppression of mHtt expression is interfered by Rad23b (N=6, one-way ANOVA, aggregates of EV+NC+G84Q versus EV+miR-196a+G84Q, P=0.0004; EV+miR-196a+G84Q versus Rad23b+miR-196a+G84Q, P=0.0060; soluble form of EV+NC+G84Q versus EV+miR-196a+G84Q, P=0.0011; EV+miR-196a+G84Q versus Rad23b+miR-196a+G84Q, P=0.0063). Empty vector (EV) and miRNA NC (NC) are served as control treatments. Significantly different at *p < 0.05; **p < 0.01

Since Rad23b influences mHTT aggregates and miR-196a directly regulates Rad23b expression, we hypothesize that Rad23b is involved in the miR-196a-mediated suppression of mHTT expression. Here, we transfected Rad23b into miR-196a/GFP-84Q cells again to block the miR-196a effects, and assessed the aggregates of GFP-84Q. Fluorescent images revealed a reduction of mHTT aggregates after the miR-196a treatment, whereas this suppressive effect was blocked by Rad23b overexpression (Fig. 4A). WB analysis further confirmed that the expression levels of the aggregated and soluble forms of mHTT were suppressed by miR-196a, whereas this effect was reversed upon Rad23b overexpression (Fig. 4B, C). These results suggested that Rad23b plays a critical role in the miR-196a-mediated suppression of mHTT aggregates.

### The interaction between mHTT and Rad23b

**Fig. 5:**
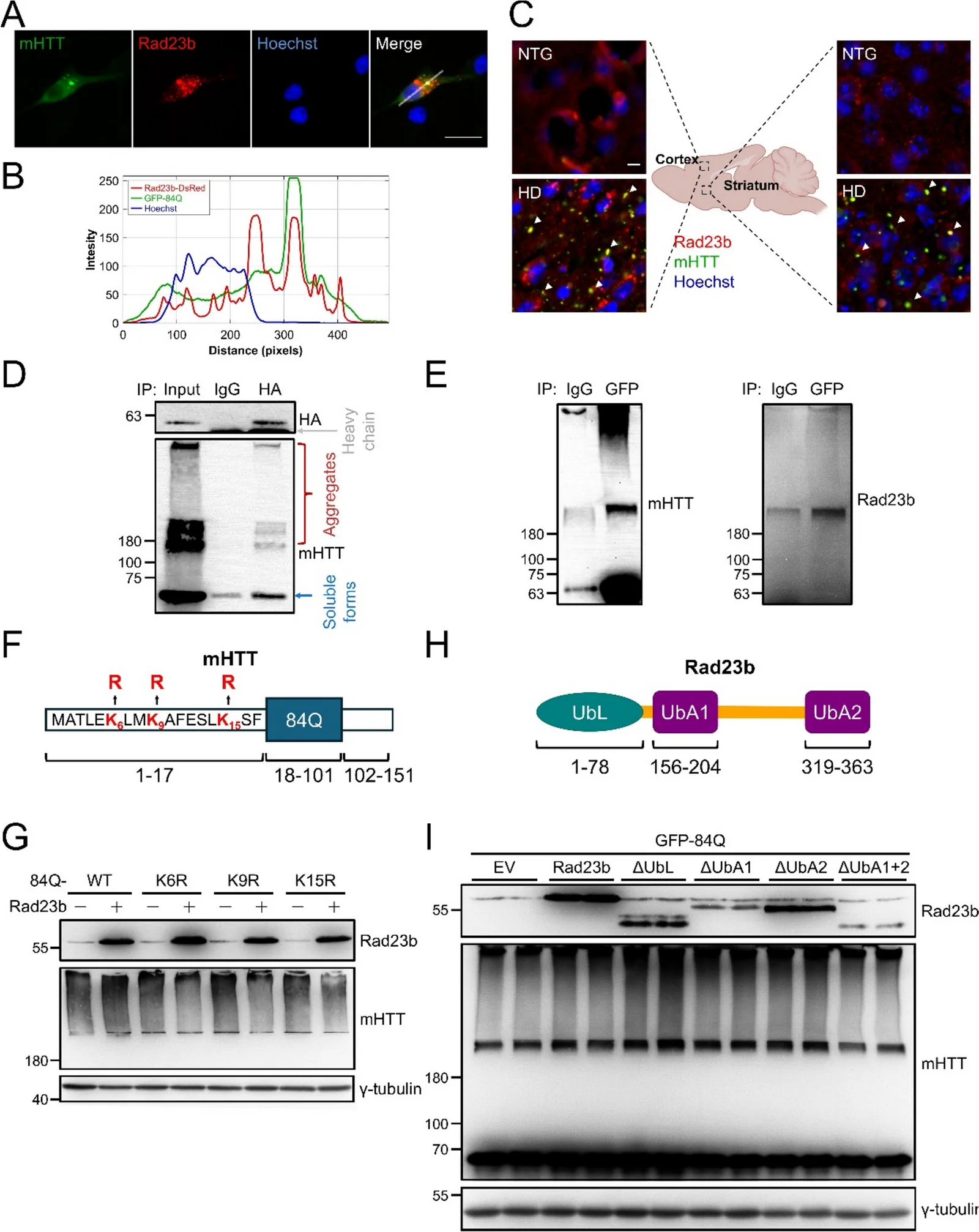
Rab23b directly interacts with mHTT. **A** Fluorescent images show the distribution of mHTT (green), DsRed-Rad23b (red), and the nucleus (blue) in N2a cells. Scale bar, 20 μm. **B** The plot profile analysis shows the signal intensity of mHTT, Rad23b, and Hoechst along the line in the transfected cell from (**A**). **C** Immunohistochemistry staining shows the distribution of mHTT and Rad23b in the cerebral cortex and striatum of non-transgenic (NTG) and HD mice at 3 months of age. Arrowheads indicate the colocalization of mHTT and Rad23b. Nuclei (blue) were stained with Hoechst 33342. Scale bar, 10 μm. **D** Co-IP analysis shows that GFP-84Q is pulled down by the HA antibody. Precipitation was performed using normal rat IgG or HA antibody. **E** A reciprocal Co-IP analysis shows that Rad23b is pulled down by the GFP antibody. **F** A scheme shows the mHTT displays 3 lysine (K) sites, which were replaced with arginine (R) in different mutant constructs. The numbers indicate the amino acid position in mHTT. **G** Immunoblots show the expression of mHTT aggregates after overexpressing Rad23b with different mHTT-84Q constructs carrying different lysine mutations. **H** A scheme shows the different domains in the Rad23b. The numbers below indicate the amino acid position in Rad23b. **I** Immunoblots show the expression of mHTT aggregates after overexpressing GFP-84Q with different truncated Rad23b constructs. Empty vector (EV) is served as a control. The Δ indicates domain deletion

Since accumulation of Rad23b has been observed in neuronal inclusions of certain neurodegenerative diseases [25], we are curious about whether Rad23b is colocalized with mHTT aggregates. We generated a DsRed-tagged Rad23b construct, and co-transected with GFP-84Q into N2a cells. Based on the fluorescent images, it showed that DsRed-tagged Rad23b forms puncta and co-localizes with GFP-84Q aggregates (Fig. 5A), which is also supported by a plot profile analysis showing the signals of DsRed-Rad23b and GFP-84Q align at the same positions along the scanned line (Fig. 5B). Additionally, we examined the distribution of endogenous Rad23b and mHTT in the cerebral cortex and striatum of HD transgenic mice, showing Rad23b forms puncta and co-localizes with mHTT aggregates in HD transgenic mice, whereas Rad23b is evenly distributed in non-transgenic (NTG) mice (Fig. 5C). Since Rad23b and mHTT colocalize together in vitro and in vivo, we next examine whether these two proteins have physically interact. *HA-tagged Rad23b* (*HA-Rad23b*) and *GFP-84Q* were co-transfected into N2a cells, and co-immunoprecipitation (Co-IP) results show both the soluble forms and aggregates of GFP-84Q are pulled down by the HA antibody, but not by the IgG control (Fig. 5D). A reciprocal CoIP was performed using a GFP antibody as well, showing Rad23b proteins are also co-precipitated and co-localized with GFP-84Q (Fig. 5E). These results support the direct interaction between Rad23b and mHTT.

Given that Rad23b functions as an ubiquitin binding protein [42] and three lysine residues (K6, K9 and K15) in the N-terminus of mHTT could potentially be ubiquitinated [43], we next try to identify the critical lysine site(s) of the N-terminus of mHTT for Rad23b-mediated regulation of mHTT. Since the GFP fragment in GFP-84Q also contains lysine residues that could interfere with the analysis, we removed this GFP fragment to eliminate this potential interference. We then generated vectors with mutations in different lysine sites in mHTT-84Q (Fig. 5F), and co-transfected these constructs with Rad23b or an empty vector into N2a cells. WB analysis revealed that mutation at each lysine site resulted in reduced mHTT-84Q aggregates (Fig. 5G). This observation was further confirmed in constructs with double and triple lysine mutations in mHTT-84Q (Supplementary Fig. 3), suggesting K6, K9 and K15 residues of mHTT-84Q are all important for Rad23b-enhanced mHTT aggregates. Next, we investigated critical domains of Rad23b responsible for interaction with mHTT, and primarily focused on the ubiquitin-related domains of Rad23b, including one UbL domain at the N-terminus and two UbA domains (UbA1 and UbA2) located in the middle and C-terminal regions (Fig. 5H). We constructed plasmids with different deletions of individual Rad23b domains (ΔUbL, ΔUbA1, ΔUbA2, or ΔUbA1+2), and transfected these truncated constructs with GFP-84Q into N2a cells. Interestingly, deletion of either UbA1 or UbA2 alone does not decrease mHTT aggregates, but deletion of both UbA domains abolishes the accumulated mHTT. Additionally, deletion of UbL region oppositely increases mHTT aggregates with known reasons (Fig. 5I). These findings suggested the importance of different ubiquitin-related domains for Rad23b to regulate mHTT aggregates.

### Rad23b regulates mHTT aggregates through the UPS pathway

**Fig. 6:**
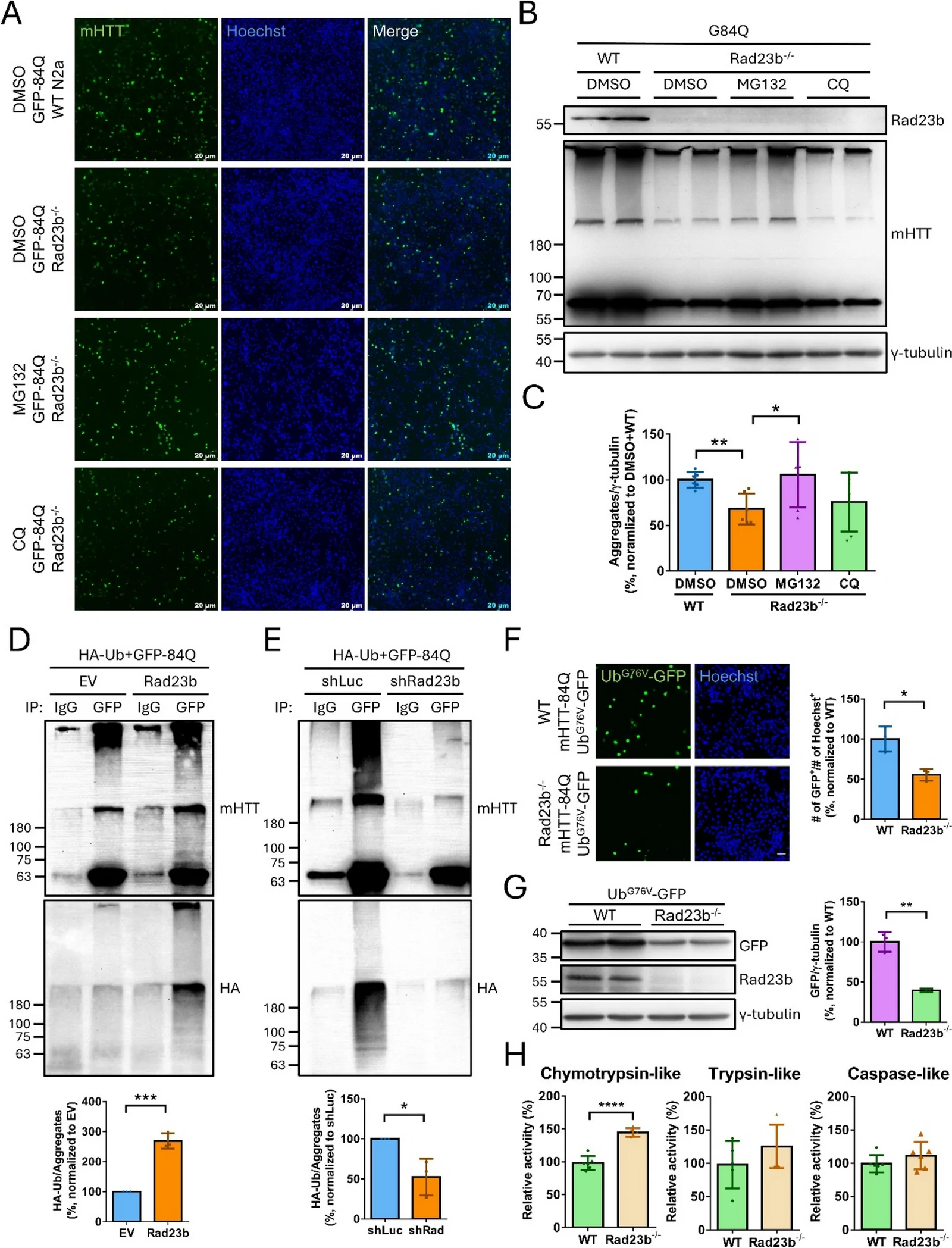
Rad23b enhances mHTT aggregates via exacerbating UPS activity. **A** Fluorescent images show the expression of GFP-84Q in Rad23b^−/−^ cells after the treatments with MG132 or chloroquine (CQ). Nuclei (blue) were stained with Hoechst 33342. **B** Immunoblots show the expression of GFP-84Q in Rad23b^−/−^ cells after treatments with MG132 or CQ. **C** The quantitative results indicate the aggregates of GFP-84Q are reversed after the MG132 treatment in Rad23b^−/−^ cells, but not after the CQ treatment (N=6, one-way ANOVA, DMSO in WT versus DMSO in Rad23b^−/−^, P=0.0021; DMSO in Rad23b^−/−^, versus MG132 in Rad23b^−/−^, P=0.0413). Significantly different at *p< 0.05; **p< 0.01. **D** CoIP was performed using cells overexpressing HA-Ub, GFP-84Q and Rad23b, and protein complex were precipitated using IgG or GFP antibodies. Immunoblots were performed using mEM48 or HA antibodies. Immunoblots (top panel) and quantitative (bottom panel) results of CoIP show Rad23b increases conjugated ubiquitin with GFP-84Q. Empty vector (EV) served as the control (two-tailed Student’s t test, P=0.0003). **E** Immunoblots (top panel) and quantitative (bottom panel) results of Co-IP show that shRad23b decreases conjugated ubiquitin with GFP-84Q. Precipitation was performed using IgG or GFP antibodies, and immunoblots were performed using mEM48 or HA antibodies (two-tailed Student’s t test, P=0.0228). **F** Fluorescent images (left panel) show Rad23b^−/−^ cells co-transfected with the Ub^G76V^-GFP and mHTT-84Q constructs. Scale bar, 50 μm. The quantitative results (right panel) indicate that the levels of Ub^G76V^-GFP are decreased in Rad23b^−/−^ cells (N=3, two-tailed Student’s t test, P=0.0111). Significantly different at *p<0.05. **(G)** Immunoblots (left panel) show the expression of Ub^G76V^-GFP in Rad23b^−/−^ N2a cells, and the quantitative results (right panel) indicate Ub^G76V^-GFP is decreased in the HD-Rad23b^−/−^ cells (N=3, two-tailed Student’s t test, P=0.0011). Significantly different at **p<0.01. **H** The chymotrypsin-like, trypsin-like and caspase-like activities are determined in WT and Rad23b^−/−^ cells transfected with GFP-84Q constructs (N=6, two-tailed Student’s t test, P for chymotrypsin-like activity<0.0001)

Rad23b functions as an ubiquitin binding protein [42], and ubiquitin is involved in regulating both UPS and autophagy for protein degradation [18]. Because Rad23b could interact with mHTT to increase mHTT aggregates, we attempted to determine which protein degradation pathway is involved in Rad23b-mediated mHTT aggregation. We transfected GFP-84Q into Rad23b^−/−^ cells, and then treated with MG132, a proteasome inhibitor, or chloroquine (CQ), an autophagy inhibitor, to examine mHTT aggregates. In results of fluorescence images, it reveals that MG132 treatment restored the number of GFP-84Q-positive cells in Rad23b^−/−^ cells, while CQ treatment had no effects on the mHTT aggregates (Fig. 6A). WB and quantitative analyses confirmed that MG132, but not CQ, restored the GFP-84Q aggregates in the Rad23b^−/−^ cells (Fig. 6B, C), suggesting Rad23b regulates GFP-84Q aggregates dominantly through the UPS pathway. Next, ubiquitination of target protein is an important process in UPS [42], and Rad23b regulates mHTT aggregates primarily through UPS in above results. Therefore, we assessed the ubiquitination levels of GFP-84Q after alteration of Rad23b. GFP-84Q and HA-tagged Ub (HA-Ub) were co-transfected into N2a cells with Rad23b or shRad23b, and Co-IP using a GFP antibody was performed to pull down mHTT complex, followed by WB using mHTT and HA antibodies. Results showed higher levels of HA-Ub signals in the GFP-84Q precipitates after Rad23b overexpression (Fig. 6D), while knockdown of Rad23b reduced the ubiquitination level of GFP-84Q (Fig. 6E). However, this is a conflict that ubiquitylated GFP-84Q proteins were increased, but the protein levels of GFP-84Q were not reduced. Therefore, we further examined the effects of Rad23b on the UPS activity using an Ub^G76V^-GFP reporter assay [44]. We co-transfected Ub^G76V^-GFP and mHTT-84Q constructs in wild-type and Rad23b^−/−^ cells, and showed that a reduced number of GFP-positive cells is detected in Rad23b^−/−^ cells (Fig. 6F), suggesting Rad23b^−/−^ cells display better proteasomal functions to degrade Ub^G76V^-GFP proteins. The results are also supported by the WB analysis, showing lower Ub^G76V^-GFP protein levels in Rad23b^−/−^ cells (Fig. 6G). In contrast, higher Ub^G76V^-GFP levels were observed after the overexpression of Rad23b in HD-N2a cells (Supplementary Fig. 4). We further determine three major catalytic activities of UPS, including chymotrypsin-like, trypsin-like, and caspase-like activities [45], in Rad23b^−/−^ HD-N2a cells, showing a significant increase in chymotrypsin-like activity in Rad23b^−/−^ cells (Fig. 6H). These results suggest that Rad23b increases mHTT aggregates dominantly through dysfunctions of UPS activity, especially chymotrypsin-like proteasome activity.

### Rad23b accelerates the onset of HD in vivo

**Fig. 7:**
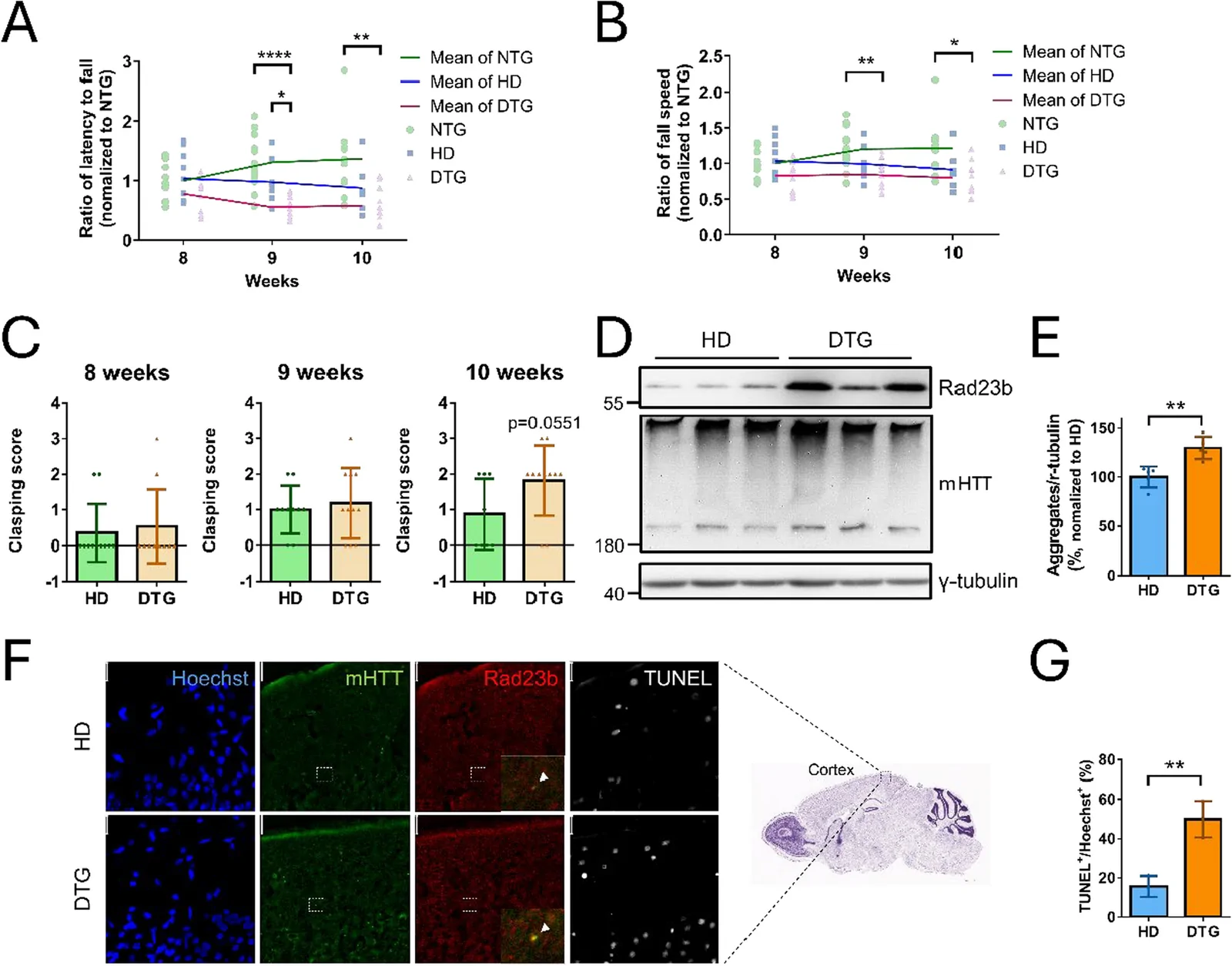
Rad23b worsens motor dysfunction and mHTT pathology in HD mice in vivo. **A**, **B** Longitudinal rotarod performance of WT, HD, and Rad23b/HD DTG mice from 8 to 10 weeks of age. Individual data points are shown to indicate biological variability among animals. Multiple comparisons were performed using Šídák’s multiple comparisons test. Significantly different at *p< 0.05; **p< 0.01; ****p< 0.0001. **C** Hindlimb clasping assay show the score of DTG mice and HD mice at different ages (two-tailed Student’s t test). **D** Immunoblots show the expression of mHTT aggregates in brains of HD and Rad23b/HD DTG mice at 6 weeks of age. **E** Quantitative results demonstrate that there are significantly more mHTT aggregates in DTG mice (N=5, two-tailed Student’s t test, P=0.0028). Significantly different at **p<0.01. **F** Immunohistostaining using anti-mHTT (green) and anti-Rad23b (red) antibodies and TUNEL assay (white) were used in the retrosplenial cortex of mice at 6 weeks of age. Nuclei (blue) were stained with Hoechst 33342. Merged images are from the squares. Arrowheads indicate mHTT aggregates. Scale bar, 20 μm. **G** Quantitative results indicate that there are more TUNEL^+^ cells in the cerebral cortex of DTG mice (N=3, two-tailed Student’s t test, P = 0.0051)

Since Rad23b impairs UPS in HD in vitro, we next demonstrate the effects of Rad23b in R6/2 HD transgenic mice in vivo. We generated Rad23b transgenic mice overexpressing Rad23b using lentiviral transgenesis [46], and crossed with R6/2 HD transgenic mice to obtain Rad23b/HD double transgenic (DTG) mice. We first evaluated the motor performance in R6/2 HD and DTG transgenic mice using the rotarod and hindlimb clasping tests. At 8 weeks of age, Rad23b/HD DTG mice exhibited a slight reduction of time on the rod, but a significant reduction in the rotational speed at which the mice fell. At 9 weeks of age, both parameters were significantly reduced in Rad23b/HD DTG mice compared to those in HD mice (Fig. 7A, B). In the hindlimb clasping test, results showed a slight increase in clasping scores in Rad23b/HD DTG mice at 8 to 10 weeks of age, indicating worsened motor function (Fig. 7C). These two motor tests suggest that Rad23b contributes to the worsening of motor dysfunction in HD.

In addition to behavioral examinations, we also demonstrated the profiling of mHTT aggregates in these mice. The cerebral cortex samples of HD and Rad23b/HD DTG mice at 6 weeks of age were used for the WB analysis (Fig. 7D), showing mHTT aggregates are elevated in the Rad23b/HD DTG mice (Fig. 7E). These samples were also subjected for pathological analysis via immunofluorescent staining (IF) using mEM48 and Rad23b antibodies, showing that more mHTT aggregates, colocalized with Rad23b, are observed in the Rad23b/HD DTG mice in retrosplenial cortex (Fig. 7F and Supplementary Fig. 5). Moreover, significantly more cell death is observed via the detection of TUNEL staining in Rad23b/HD DTG mice (Fig. 7G and Supplementary Fig. 5). These results are also shown in the motor cortex and striatum of HD and Rad23b/HD DTG mice (Supplementary Fig. 6). Moreover, we also examined whether Rad23b or miR-196a transgenic mice, two control mouse lines, alone exhibit HD-like neuropathological features in the motor cortex and striatum. Results show that both Rad23b and miR-196a transgenic mice did not show obvious mHTT aggregation or TUNEL-positive pathology (Supplementary Fig. 7), suggesting that Rad23b or miR-196a transgene expression alone does not recapitulate the neuropathological features of R6/2 HD mice. To further examine whether Rad23b affects proteasome function in vivo, we measured the three major proteasomal catalytic activities in cerebral cortex lysates from HD and Rad23b/HD DTG mice. Rad23b/HD DTG mice showed a tendency toward reduced chymotrypsin-like, trypsin-like, and caspase-like activities compared with HD mice; however, these differences did not reach statistical significance (Supplementary Fig. 8). These results suggest Rad23b worsens motor behaviors and accelerates mHTT aggregates and cell death in HD transgenic mice in vivo.

## Discussion

HD is a terrible neurodegenerative disease, and there is no cure now, resulting in patients bring huge burdens for their families and modern society. miR-196a is a potential target that improves the phenotypes of HD in several different models [15]; however, due to no miR-196a binding sites in *mHTT* gene, how miR-196a downregulates the mHTT aggregates is still completely unclear yet. Here, we identified Rad23b as a new target of miR-196a, and miR-196a could suppress mHTT aggregates through inhibition of Rad23b. Rad23b not only interacts with mHTT, but also downregulates UPS activity and leads to accumulation of mHTT aggregates. Moreover, Rad23b also shows to worsen HD characteristics in HD transgenic mice in vivo, highly suggesting the potential application of miR-196a in the therapy of HD.

miR-196a has been reported to involve in different diseases, such as cancers [47], chronic kidney disease [48], endometriosis [49], etc. It targets different genes to regulate cell proliferation, migration [50], differentiation [51]. In HD, miR-196a ameliorates several phenotypes in different HD models through promoting neuronal morphology in HD via RANBP10 [13] and insulin-like growth factor 2 mRNA binding protein 3 (IGF2BP3) [14], providing antioxidative functions via USP15 [14], etc. In this study, we further validate that miR-196a improves UPS activity to decrease mHTT aggregates. These results suggest miR-196a may provide beneficial function through different mechanisms, resulting in better neuroprotective effects in HD.

In addition to the above mechanisms, the cellular metabolism is also a critical process to maintain physiological conditions and to prevent disease progression. In neurodegenerative diseases, the dysfunction of metabolism in central or peripheral systems has been reported, leading to abnormality of neuronal activity [52]. Upon results in our proteomic analysis (Fig. 1B), biosynthesis of amino acids, cysteine and methionine metabolism, autophagy, AMPK signaling pathways and 2-oxocarboxylic acid metabolism are upregulated by the miR-196a treatment, implying that miR-196a may facilitate resource conversation, generate energy and recycle internal components to sustain essential functions of neuronal metabolism under the HD condition. Therefore, how miR-196a regulates downstream targets to further influence neuronal metabolism is still needed to be demonstrated.

In addition to upregulated pathways, we also found miR-196a downregulates several target proteins related to the pathways of neuronal diseases, such as ALS, prion disease, HD, Alzheimer disease, Parkinson’s disease and pathways of neurodegeneration. Echoing with the previous microarray and bioinformatic analyses in HD-miR-196a transgenic mice [53], it highly suggests miR-196a may have pan-effects on different neuronal diseases, which shares common pathological characteristics such as oxidative stress, mitochondrial dysfunction, aggregate formation, etc. [54]. Especially, a prion disease, which is caused by protein misfolding in central nerve system [55], has been strongly linked to the results of above high-through put screening. Since we have addressed the beneficial effects of miR-196a on mHTT aggregates in this study, it also suggests miR-196a may influence the protein misfolding of prion disease, which has not been reported yet. It would be interesting to further demonstrate the effects of miR-196a in this protein misfolding disease.

Rad23b functions as an ubiquitin binding protein [42], and we showed Rad23b directly interacts with mHTT (Fig. 5). Most interestingly, Rad23b also colocalizes with pathological aggregates in FTD, FTDP-17, ALS and Parkinson's disease [24–26], suggesting Rad23b may have certain pan-effects related to UPS in different aggregate diseases. Although above studies did not further confirm the direct interaction between aggregate proteins and Rad23b, these results still raise an interesting issue regarding the role of Rad23b in these aggregate proteins. Since elimination of aggregates is one of the therapeutical directions in these neuronal diseases, further investigations on the interaction between Rad23b and disease-aggregates would be important.

Additionally, we showed Rad23b increases mHTT aggregates in vitro and in vivo (Fig. 3 and 7), indicating higher expression of Rad23b exacerbates HD. However, Rad23b has been reported to offer the beneficial effects in other diseases. For example, Rad23b has been reported to be sequestrated in poly-Glycine-Alanine (poly-GA) proteins-positive inclusions in *C9ORF72*-linked FTD and ALS [24, 56, 57], and restoring Rad23b levels attenuates poly-GA aggregates and poly-GA-induced toxicity [24]. This result could be interpreted upon that Rad23b is considered as a ubiquitin shuttle protein [58, 59], and the sequestration of Rad23b decreases the shuttle function to proteasomes, leading to accumulation of aggregates. These conflicting results suggest the complicated role of Rad23b in different aggregate proteins; especially the enriched interacting proteins in different aggregates are different [60]. Therefore, we speculate the working mechanism of miR-196a through suppression of Rad23b is relatively fit for polyQ aggregates; however, further evidence is needed to be provided.

Due to the function of Rad23b, we explained Rad23b regulates mHTT aggregates through regulation of UPS. Although our cell-based experiments showed that Rad23b affects UPS function (Fig. 6H), the in vivo proteasome activity assay using cortical lysates did not reveal a statistically significant reduction in chymotrypsin-like activity in Rad23b/HD DTG mice (Supplementary Fig. 8). One possible explanation is that whole cortical lysates contain mixed cell populations, including neurons and non-neuronal cells. Therefore, the measured proteasome activity represents the averaged activity of the entire tissue lysate and may not fully reflect neuron-specific proteasomal impairment. In addition, region-specific or cell type-specific changes may be diluted or masked in bulk cortical lysates. Future studies using purified neuronal populations, laser-capture microdissection, or cell type-specific approaches will be required to determine whether Rad23b selectively alters neuronal proteasome activity in vivo. Furthermore, a limitation for this aspect is that we only focused on the role of Rad23b in this pathway. However, the aggregate formation is a complicated process. We believe that UPS regulation is not the only one pathway that miR-196a regulates based on the microarray and bioinformatic analysis in miR-196a/HD DTG mice [53]. In addition, a new function of Rad23b was identified in histone demethylation [61]. It indicates that Rad23b is also involved in gene transcription. Therefore, future studies should investigated the different aspect role of Rad23b in the formation of mHTT aggregates.

A limitation of the present study is that behavioral analyses were focused on motor-related assays, including rotarod and hindlimb clasping tests (Fig. 7A-C). Although these tests are commonly used as representative behavioral readouts in R6/2 and other HD mouse studies, they do not fully cover the broad clinical manifestations of HD, such as cognitive dysfunction, spontaneous activity changes, and chorea-like movements. Future studies using additional behavioral paradigms, including beam walking, open field, and Y-maze or other spatial recognition tests, will be needed to determine whether Rad23b also affects non-motor and cognitive phenotypes in HD mice. Another limitation is that we only showed Rad23b and miR-196a transgenic mice did not exhibit obvious HD-like mHTT aggregation or TUNEL-positive pathology in the motor cortex or striatum without systematic behavioral assays in the present study (Supplementary Fig. 7). To fully define their phenotypic profiles, future studies with systematic behavioral assays will be needed to fully characterize subtle behavioral phenotypes in these single-transgenic lines.

## Conclusions

Taken together, we show Rad23b not only accelerates the onset of HD but also functions as a molecular hub linking miR-196a, ubiquitination, and protein aggregation. These results suggest that downregulating Rad23b expression or disrupting its interaction with mHTT may offer novel strategies to mitigate mHTT aggregation and delay HD progression, even applying to other polyQ diseases.

## Supplementary Information

Supplementary Material 1

## Data Availability

No datasets were generated or analysed during the current study.

## Abbreviations

3’UTR: 3’ Untranslated region
ALS: Amyotrophic lateral sclerosis
AMC: 7-Amino-4-methylcoumarin
ANOVA: Analysis of variance
ATXN3: Ataxin 3
C9ORF72: Chromosome 9 open reading frame 72
CMV: Cytomegalovirus
Co-IP: Co-immunoprecipitation
CQ: Chloroquine
DAVID: Database for Annotation, Visualization and Integrated Discovery
DTG: Double transgenic
EGFP: Enhanced green fluorescent protein
FTD: Frontotemporal dementia
FTDP-17: Frontotemporal dementia with parkinsonism-17
GEO: Gene Expression Omnibus
GFP-84Q: mHTT-containing 84Q fused with GFP
HA: Hemagglutinin
HA-Rad23b: HA-tagged Rad23b
HA-Ub: HA-tagged Ub
HD: Huntington’s disease
HTT: Huntingtin
IF: Immunofluorescence
IGF2: Insulin like growth factor 2
IGF2BP3: Insulin-like growth factor 2 mRNA binding protein 3
mATXN3: Mutant Ataxin 3
mHTT: Mutant huntingtin
miRNA: MicroRNA
NC: Non-relevant control
NER: Nucleotide excision repair
NRF2: Nuclear factor erythroid 2
NTG: Non-transgenic
PBS: Phosphate-buffered saline
PFA: Paraformaldehyde
PI: Propidium iodide
poly-GA: Poly(Glycine-Alanine)
polyQ: Poly-glutamine
Rad23b: RAD23 homolog B, nucleotide excision repair protein
RANBP10: RAN binding protein 10
ROI: Region of interest
rpm: Revolutions per minute
SCA3: Spinocerebellar ataxia type 3
SD: Standard deviation
SDS-PAGE: Sodium dodecyl sulfate polyacrylamide gel electrophoresis
shLuc: ShRNA of luciferase
Ub: Ubiquitin
UbA: Ubiquitin-associated
UbL: Ubiquitin-like
UPS: Ubiquitin-proteasome system
USP15: Ubiquitin-specific peptidase 15
WB: Western blotting
