# Supplementary material for: miR-196a reduces mutant Huntingtin aggregates by Rad23b-mediated degradation in Huntington’s disease
Source: J Biomed Sci. 2026 Sep 24;33:90. doi: 10.1186/s12929-026-01292-5 (PMC13613857; doi:10.1186/s12929-026-01292-5)
Supplement: Supplementary file 1 [file 12929_2026_1292_MOESM1_ESM.pdf]

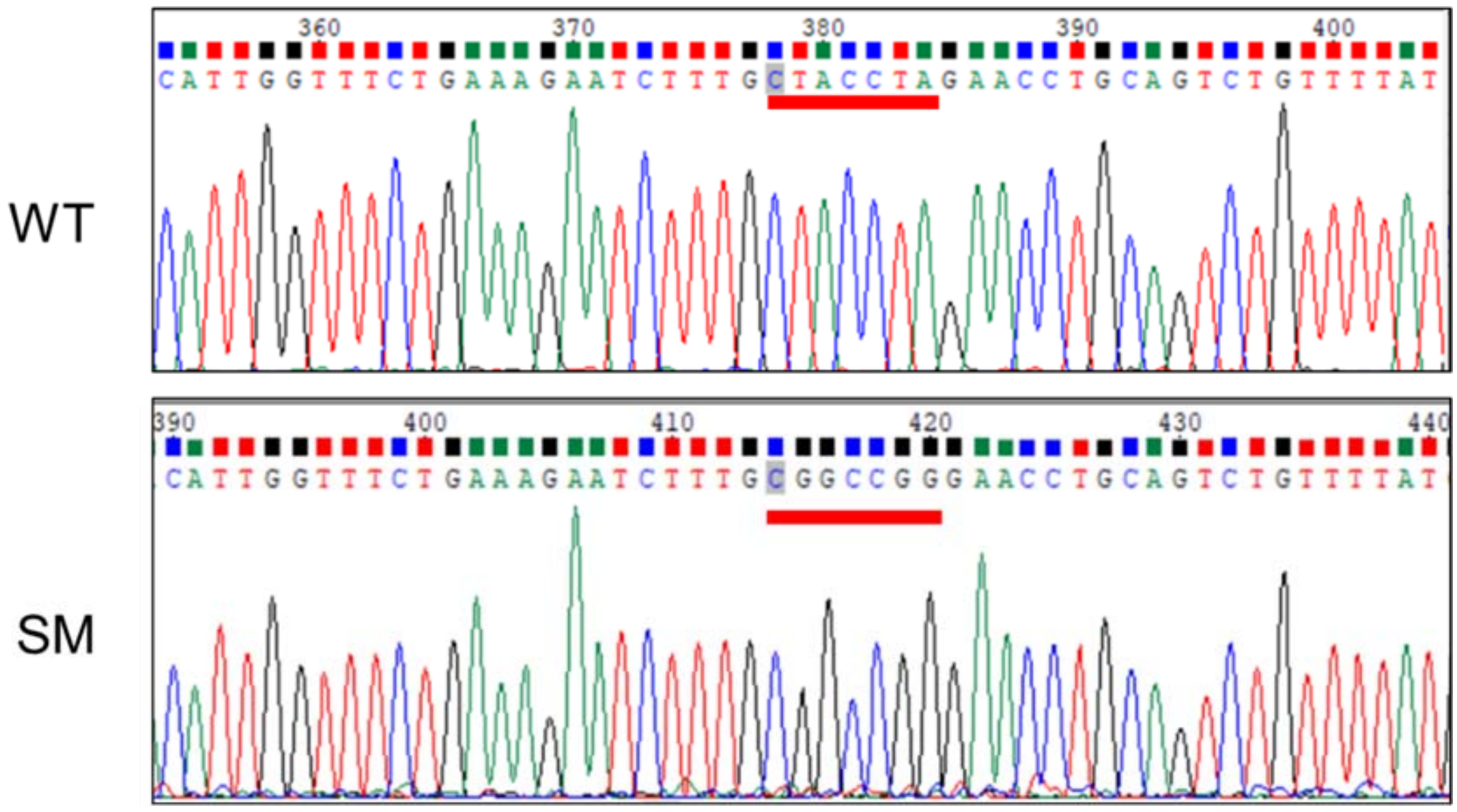

Supplementary Fig. 1 Mutation of Rad23b 3'UTR in the predicted sequence of miR-196a binding site. The top panel shows the wild type sequence of miR-196a binding site in Rad23b 3'UTR and the bottom panel shows site-directed mutagenesis (SM; CTACCTA → CggCCgg) of the binding site.

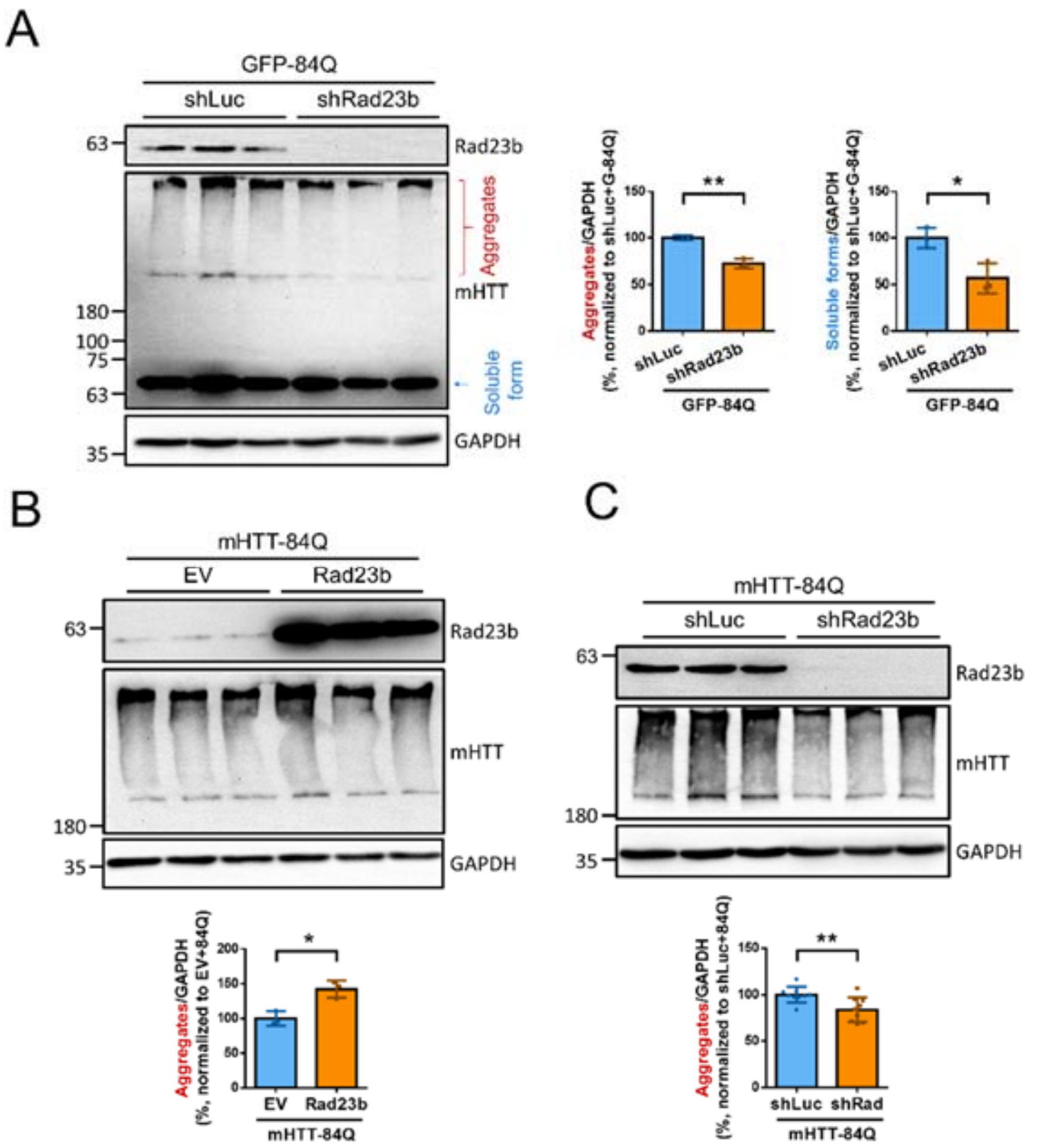

Supplementary Fig. 2 Manipulation of Rad23b affects the aggregation of mHtt protein.

(A) Immunoblots and quantitative results show the soluble form and aggregate levels of GFP-84Q are downregulated after knockdown of Rad23b (N=3, two-tailed Student's t test, P for aggregate expression = 0.0011; P for soluble form = 0.0180). **(B)** Immunoblots and quantitative results show the aggregate levels of mHTT-84Q are increased by the overexpression of Rad23b (N = 3, two-tailed Student's t test, P for aggregate expression = 0.0106). **(C)** Immunoblots and quantitative results show the aggregate levels of mHTT-84Q are decreased after knockdown of Rad23b (N = 3, two-tailed Student's t test, P for aggregate expression = 0.0079). Significantly different at *p < 0.05; **p < 0.01.

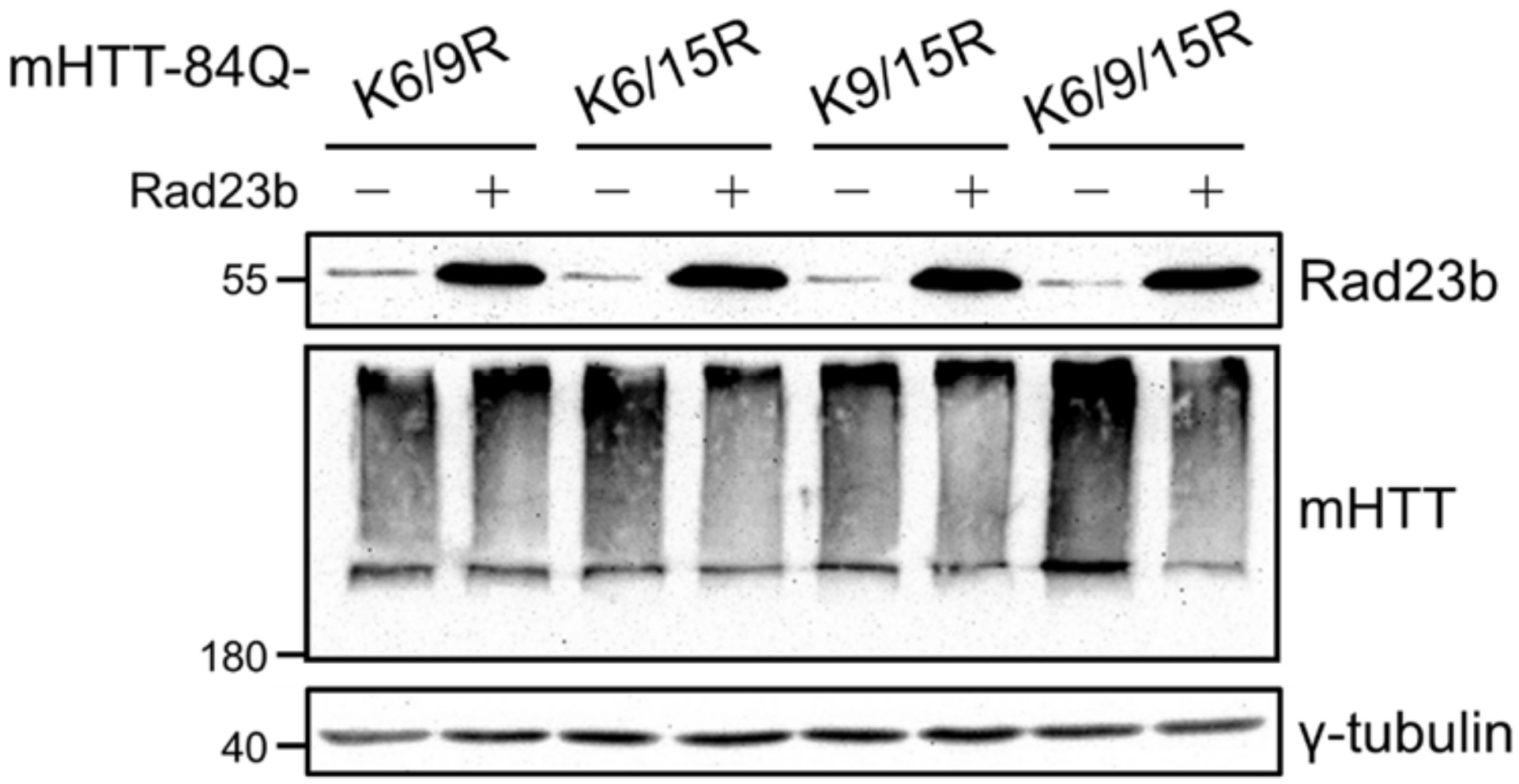

Supplementary Fig. 3 Multiple lysine mutations in mHTT affects Rad23b-enhanced mHTT aggregates. Immunoblots show the expression of mHTT aggregates after overexpressing Rad23b with different mHTT-84Q constructs carrying multiple lysine mutations as indicated.

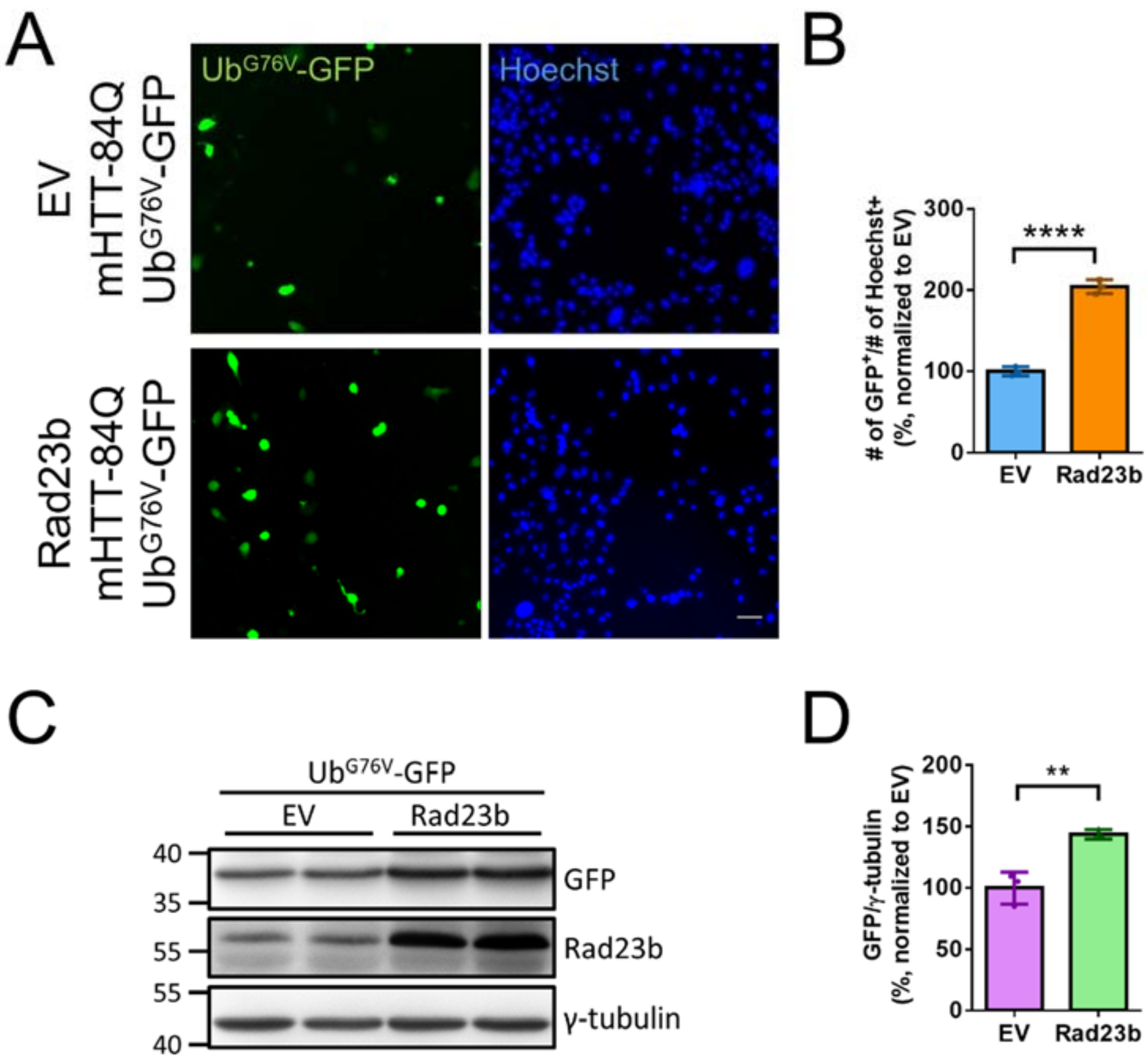

Supplementary Fig. 4 Rad23b increases the accumulation of Ub^{G76V}-GFP. Fluorescent images show N2a cells co-transfected with the Ub^{G76V}-GFP, mHTT-84Q, and either empty vector (EV) or Rad23b constructs. Scale bar, 50 µm. **(B)** Quantitative results indicate that the increased levels of Ub^{G76V}-GFP in HD-N2a cells after Rad23b overexpression (N = 3, two-tailed Student's t test, $P < 0.0001$). Significantly different at ****$p < 0.0001$. **(C)** Immunoblots show the expression of Ub^{G76V}-GFP in N2a cells after Rad23b overexpression. **(D)** Quantitative results indicate that the Ub^{G76V}-GFP is increased by Rad23b in the HD-N2a cells (N = 3, two-tailed Student's t test, $P = 0.0047$).

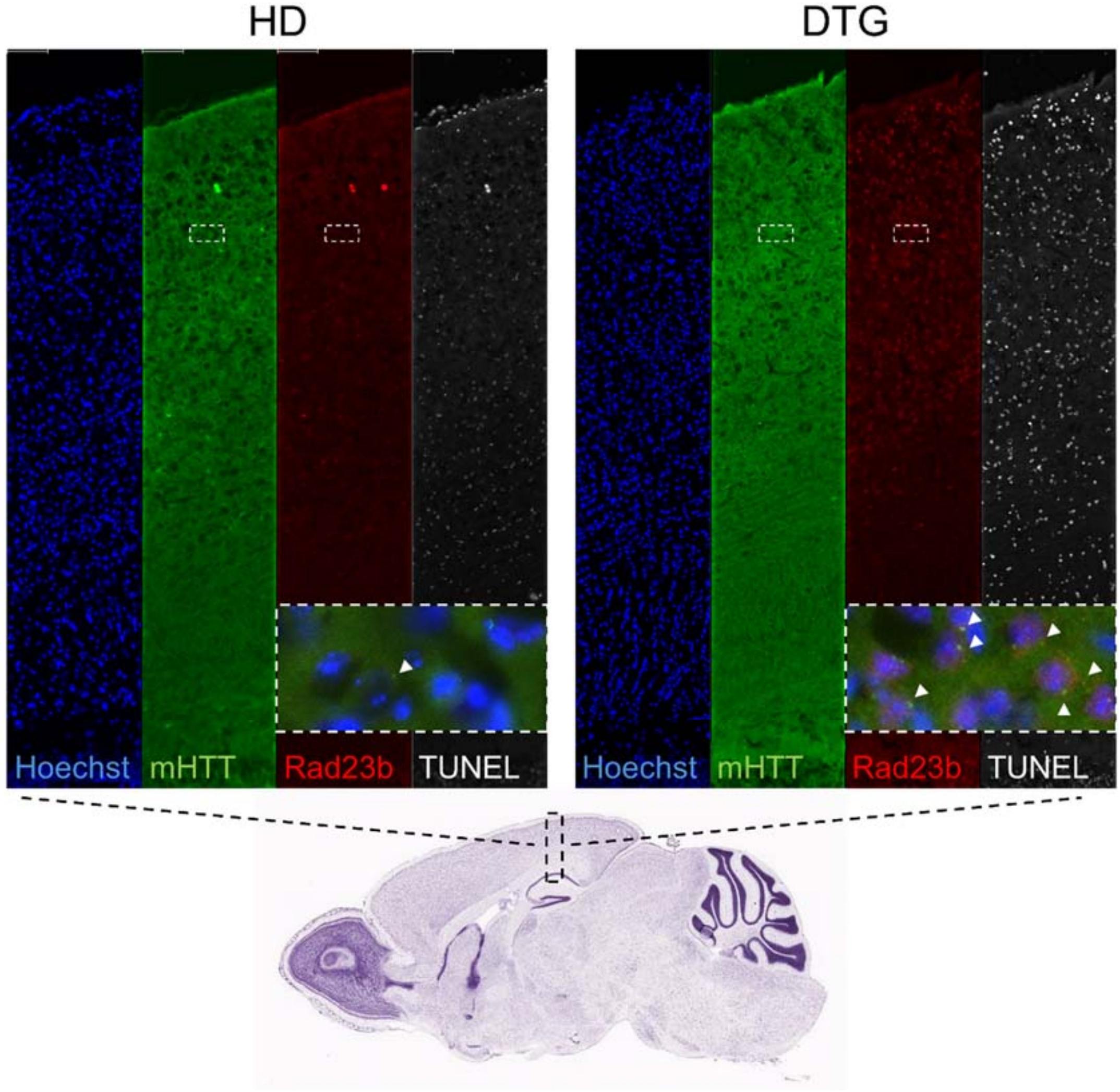

Supplementary Fig. 5 The pathological phenotypes in cerebral cortex of HD and DTG mice. Brain sections from HD and DTG mice at 6 weeks of age were subjected to TUNEL assay(red) and immunolabeling with anti-mHTT(green) and anti-Rad23b(red) antibodies. Nuclei (blue) were stained with Hoechst 33342. Scale bar, 20 μm.

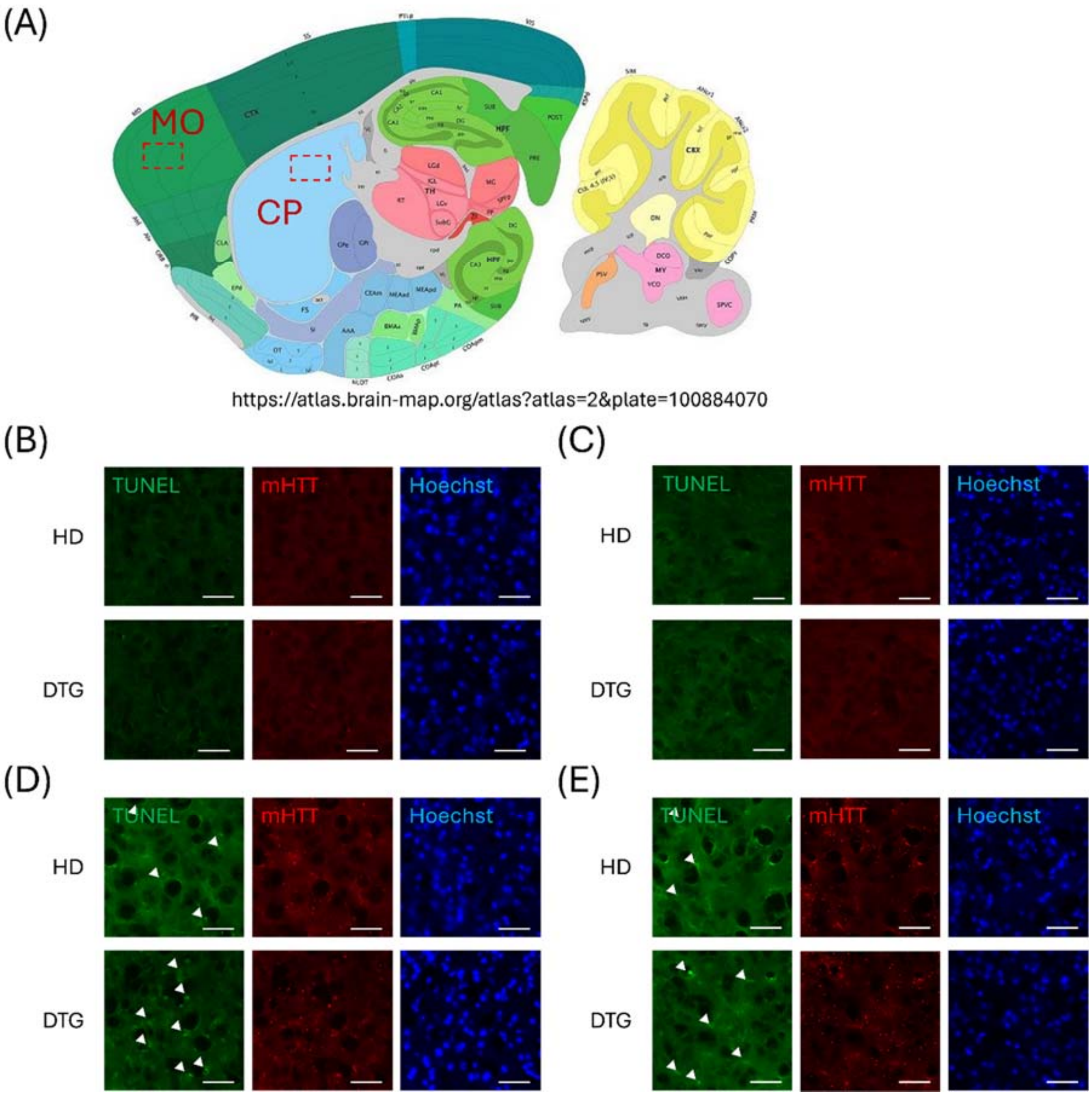

Supplementary Fig. 6 Pathological examination of the motor cortex and striatum in HD and Rad23b/HD DTG mice. (A) Schematic illustration showing the examined brain regions. Red dashed boxes indicate the motor cortex (MO) and striatum/caudate-putamen (CP) regions selected for imaging. **(B)** Representative images of TUNEL staining, mHTT immunoreactivity, and Hoechst nuclear staining in the motor cortex of 1-month-old mice. **(C)** Representative images of the striatum of 1-month-old mice. **(D)** Representative images

of the motor cortex of 2-month-old mice. **(E)** Representative images of the striatum of 2-month-old mice. Arrowheads indicate TUNEL-positive cells. Scale bar, 50 μm.

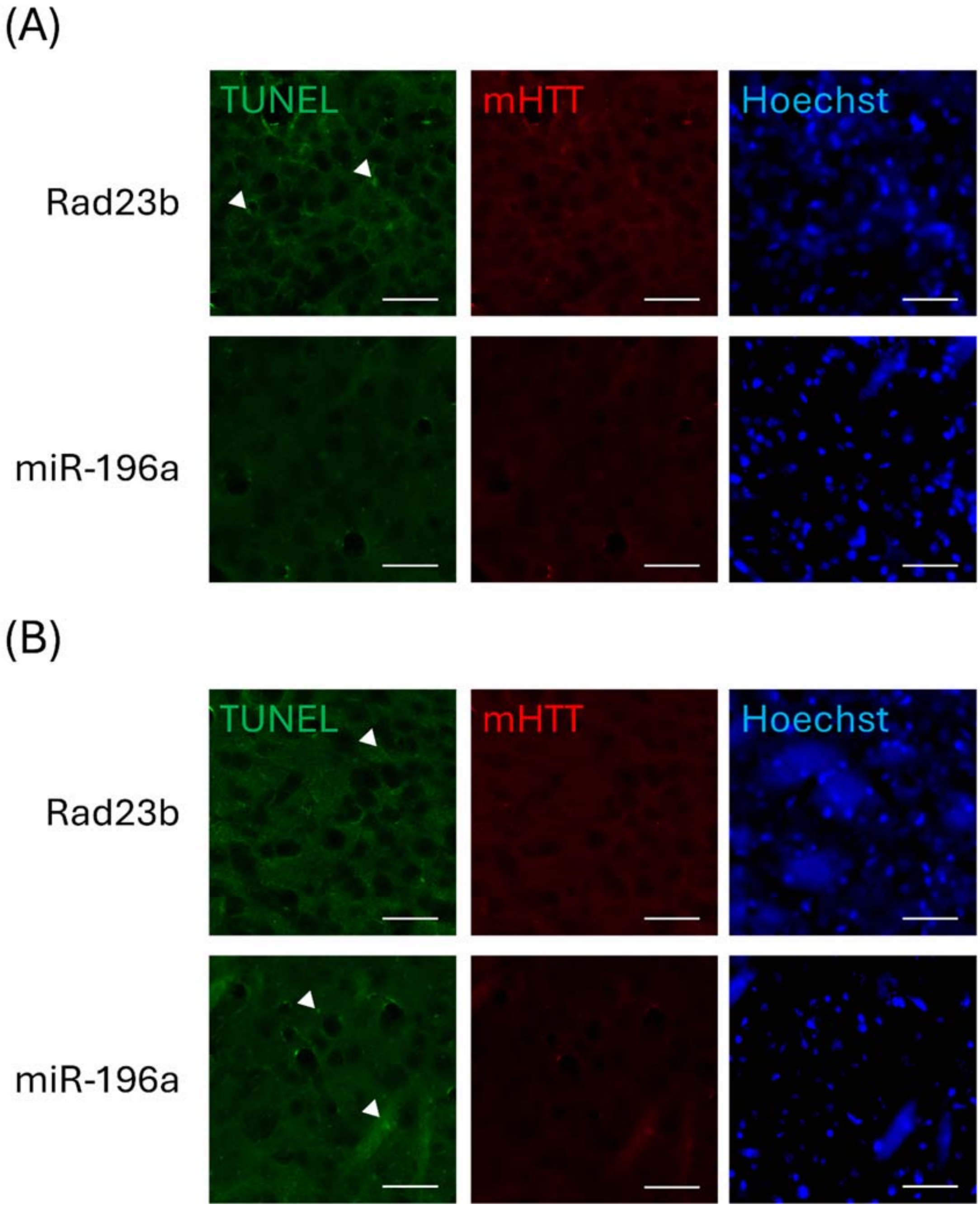

Supplementary Fig. 7 Pathological examination of HD, Rad23b transgenic, and miR-196a transgenic mice. Representative images show TUNEL staining, mHTT immunoreactivity, and Hoechst nuclear staining in the (A) motor cortex and (B) striatum of HD, Rad23b transgenic, and miR-196a transgenic mice. HD mice were examined at 2.5

months of age, Rad23b transgenic mice at 3 months of age, and miR-196a transgenic mice at 18 months of age. Arrowheads indicate TUNEL-positive cells. Scale bar, 50 μm.

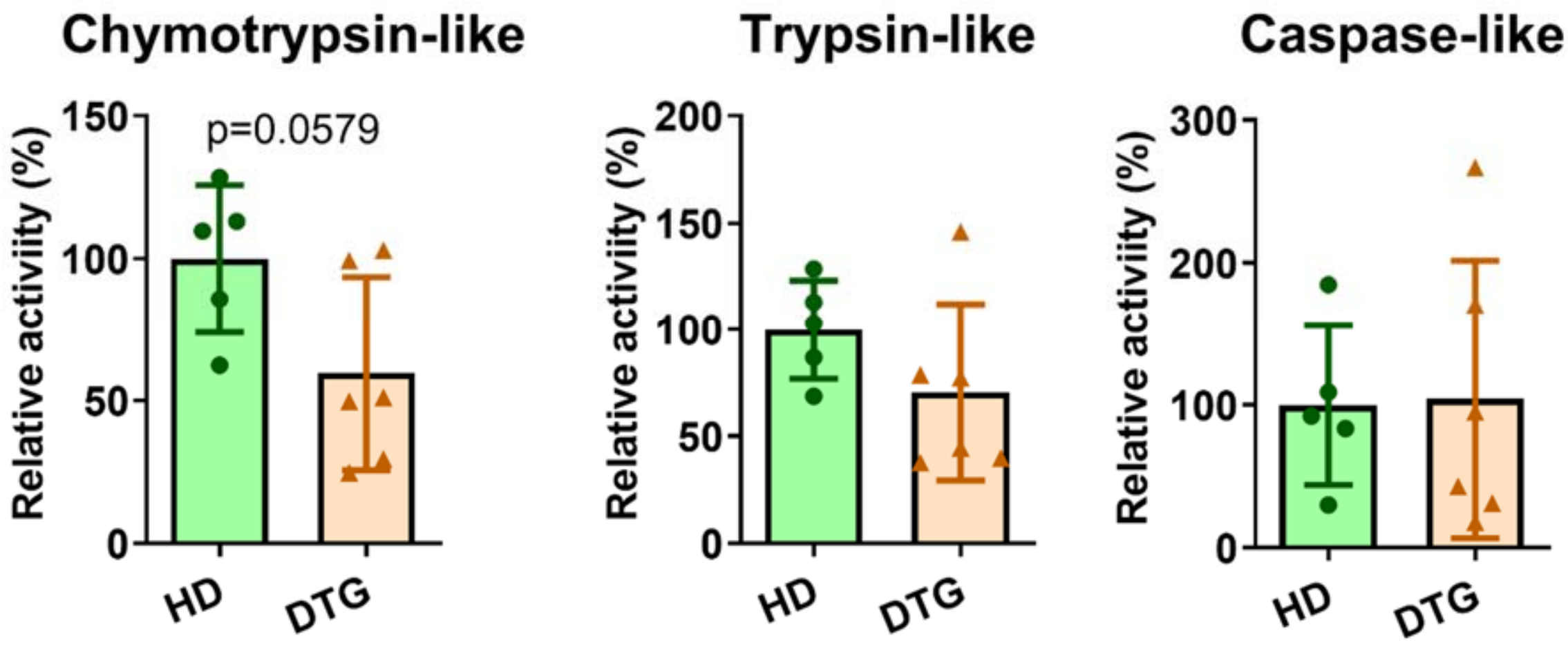

Supplementary Fig. 8 Proteasome activity assay in the cerebral cortex of HD and Rad23b/HD DTG mice. Proteasomal catalytic activities were measured in cerebral cortex lysates from HD and Rad23b/HD DTG mice using fluorogenic peptide substrates. Chymotrypsin-like, trypsin-like, and caspase-like activities were analyzed. Individual data points are shown to indicate biological variability among animals. Data are presented as mean ± SD. HD, n = 5; Rad23b/HD DTG, n = 6. Statistical analysis was performed using two-tailed Student's t test.

Supplementary Table 1. List of KEGG pathways enriched by miR-196a-upregulated proteins discovered through proteomic analysis in HD-N2a cells.

Term	p Value	Genes
DNA replication	4.24E-06	Rfc3, Mcm7, Lig1, Rpa1, Mcm3, Mcm4
Biosynthesis of amino acids	1.58E-04	Gpt2, Idh1, Taldo1, Mat2b, Bcat1, Pfkp
Mismatch repair	5.02E-04	Rfc3, Msh2, Lig1, Rpa1
Cysteine and methionine metabolism	5.43E-03	Ahcyl1, Sms, Mat2b, Bcat1
Autophagy - animal	6.04E-03	Rptor, Ppp2ca, Prkaa1, Rheb, Rras2, Prkacb
AMPK signaling pathway	9.84E-03	Rptor, Ppp2ca, Prkaa1, Rheb, Pfkp
Insulin signaling pathway	1.49E-02	Rptor, Prkaa1, Prkar1a, Rheb, Prkacb
2-Oxocarboxylic acid metabolism	2.16E-02	Gpt2, Idh1, Bcat1
Cushing syndrome	2.19E-02	Gnaq, Cdk2, Aip, Ash2l, Prkacb
Thermogenesis	2.26E-02	Rptor, Prkaa1, Rheb, Acsl4, Prkacb, Atp5mf
Cell cycle	2.33E-02	Ppp2ca, Mcm7, Cdk2, Mcm3, Mcm4
Longevity regulating pathway	2.40E-02	Rptor, Prkaa1, Rheb, Prkacb
Glucagon signaling pathway	3.74E-02	Prkaa1, Gnaq, Prkacb, Pfkp
Nucleocytoplasmic transport	3.83E-02	Ipo8, Nup107, Nup43, Rangap1
Carbon metabolism	4.57E-02	Gpt2, Idh1, Taldo1, Pfkp
Longevity regulating pathway - multiple species	6.80E-02	Rptor, Prkaa1, Prkacb
Nucleotide excision repair	7.00E-02	Rfc3, Lig1, Rpa1
Measles	7.08E-02	Cdk2, Rab9a, Eif2ak2, Adar
Apelin signaling pathway	7.21E-02	Prkaa1, Gnaq, Rras2, Prkacb
Human papillomavirus infection	7.90E-02	Itgb1, Ppp2ca, Rheb, Cdk2, Eif2ak2, Prkacb
Cellular senescence	9.40E-02	Rbbp4, Rheb, Cdk2, Rras2

Supplementary Table 2. List of KEGG pathways enriched by miR-196a-downregulated proteins discovered through proteomic analysis in HD-N2a cells.

Term	PValue	Genes
Bacterial invasion of epithelial cells	3.82E-05	Septin2, Arpc5l, Cltb, Wasl, Wasf1, Crkl, Dnm2
Oxidative phosphorylation	1.23E-04	Ndufb7, Ndufa4, Ndufab1, Ndufb1, Uqcrc2, Cox6c, Atp5f1c, Atp6v1d
Amyotrophic lateral sclerosis	1.85E-04	Ppp3r1, Ndufb7, Ndufa4, Fus, Ndufab1, Nup62, Dctn3, Ndufb1, Srsf3, Uqcrc2, Cox6c, Atp5f1c
Prion disease	3.96E-04	Ppp3r1, Ndufb7, Csnk2a1, Ndufa4, Ndufab1, Csnk2b, Ndufb1, Uqcrc2, Cox6c, Atp5f1c
Chemical carcinogenesis - reactive oxygen species	4.72E-04	Ndufb7, Ndufa4, Ndufab1, Ndufb1, Grb2, Uqcrc2, Cox6c, Sod2, Atp5f1c
Pathways of neurodegeneration - multiple diseases	4.72E-04	Ndufb7, Csnk2a1, Ndufa4, Fus, Dctn3, Ndufb1, Cox6c, Atp5f1c, Ppp3r1, Ndufab1, Csnk2b, Fadd, Uqcrc2
Thermogenesis	6.11E-04	Ndufb7, Ndufa4, Ndufab1, Ndufb1, Grb2, Uqcrc2, Cox6c, Arid1a, Atp5f1c
Huntington disease	8.90E-04	Ndufb7, Ndufa4, Ndufab1, Dctn3, Cltb, Ndufb1, Uqcrc2, Cox6c, Sod2, Atp5f1c
Shigellosis	8.93E-04	Capns1, Septin2, Arpc5l, Ube2v2, H3c1, Wasl, Ube2v1, Wasf1, Crkl
Fc gamma R-mediated phagocytosis	1.18E-03	Marcksl1, Gsn, Arpc5l, Wasf1, Crkl, Dnm2
Alzheimer disease	1.18E-03	Ppp3r1, Ndufb7, Csnk2a1, Ndufa4, Ndufab1, Csnk2b, Ndufb1, Fadd, Uqcrc2, Cox6c, Atp5f1c
Diabetic cardiomyopathy	1.34E-03	Ndufb7, Ndufa4, Ndufab1, Ndufb1, Uqcrc2, Pdhb, Cox6c, Atp5f1c
Citrate cycle (TCA cycle)	1.80E-03	Pc, Aco1, Dlat, Pdhb

Chronic myeloid leukemia	3.50E-03	Ctbp2, Cdk4, Ctbp1, Grb2, Crkl
Pyruvate metabolism	6.50E-03	Pc, Acyp1, Dlat, Pdhb
Spliceosome	8.48E-03	Sf3b4, Rbm8a, Snrpd2, Fus, Srsf2, Srsf3, Snrpa
Non-alcoholic fatty liver disease	8.92E-03	Ndufb7, Ndufa4, Ndufab1, Ndufb1, Uqcrc2, Cox6c
Parkinson disease	2.27E-02	Ndufb7, Ndufa4, Ndufab1, Ndufb1, Uqcrc2, Cox6c, Atp5f1c
2-Oxocarboxylic acid metabolism	2.94E-02	Aco1, Dlat, Pdhb
Regulation of actin cytoskeleton	3.91E-02	Enah, Gsn, Arpc5l, Wasl, Wasf1, Crkl
Adherens junction	3.99E-02	Csnk2a1, Csnk2b, Wasl, Wasf1
Metabolic pathways	4.12E-02	Pdhx, Ndufb7, Ndufa4, Ndufb1, Txndc12, Acyp1, Pdhb, Cox6c, Atp5f1c, Hadhb, Pc, Scp2, Glb1, P4ha2, Ndufab1, Aco1, Dlat, Uqcrc2, Atp6v1d, Nit2
Wnt signaling pathway	5.31E-02	Ppp3r1, Ctbp2, Csnk2a1, Ctbp1, Csnk2b
Salmonella infection	5.33E-02	Dynlt1, Dctn3, Arpc5l, Wasl, Fadd, Dnm2
Endocytosis	5.41E-02	Snx3, Hgs, Arpc5l, Cltb, Wasl, Dnm2
Carbon metabolism	6.85E-02	Pc, Aco1, Dlat, Pdhb

Supplementary table 3. List of putative miR-196a target genes

Gene symbol	Full name	Fold change*	KEGG BRITE
Rfc3	replication factor C subunit 3	2.81	DNA replication proteins<br>Chromosome and associated proteins<br>DNA repair and recombination proteins
Letmd1	LETM1 domain containing 1	0.38	No information
Slc25a22	solute carrier family 25 member 22	0.33	Transporters
Rad23b	RAD23 homolog B, nucleotide excision repair protein	0.31	Proteasome<br>DNA repair and recombination proteins
Snap91	synaptosome associated protein 91	0.25	Membrane trafficking
Larp4	La ribonucleoprotein 4	0.22	Messenger RNA biogenesis

*Direction of comparison: miR-196a/miR-NC
